# It matters how we measure - Quantification of microplastics in drinking water by μFTIR and μRaman

**DOI:** 10.1016/j.heliyon.2023.e20119

**Published:** 2023-09-13

**Authors:** L. Maurizi, L. Iordachescu, I.V. Kirstein, A.H. Nielsen, J. Vollertsen

**Affiliations:** a Department of the Built Environment, Aalborg University, 9220, Aalborg, Denmark; b Alfred-Wegener-Institute Helmholtz Centre for Polar and Marine Research, Biologische Anstalt Helgoland, Helgoland, Germany

**Keywords:** Microplastics, Raman micro-spectroscopy, FTIR micro-spectroscopy, Drinking water

## Abstract

The water treatment for microplastics (MP) at a Danish groundwater-based waterworks was assessed by Fourier-Transform IR micro-spectroscopy (μFTIR) (nominal size limit 6.6 μm) and compared to results from Raman micro-spectroscopy (μRaman) (nominal size limit 1.0 μm) on the same sample set. The MP abundance at the waterworks' inlet and outlet was quantified as MP counts per cubic metre (N/m^3^) and estimated MP mass per cubic metre (μg/m^3^). The waterworks' MP removal efficiency was found to be higher when analysing by μFTIR (counts: 78.14 ± 49.70%, mass: 98.73 ± 11.10%) and less fluctuating than when using μRaman (counts: 43.2%, mass: 75.1%). However, both techniques pointed to a value of ∼80% for the counts' removal efficiency of MPs >6.6 μm. Contrarily to what was shown by μRaman, no systematic leaking of MPs from the plastic elements of the facility could be identified for the μFTIR dataset, either from the counts (inlet 31.86 ± 17.17 N/m^3^, outlet 4.98 ± 2.09 N/m^3^) or mass estimate (inlet 76.30 ± 106.30 μg/m^3^, outlet 2.81 ± 2.78 μg/m^3^). The estimation of human MP intake from drinking water calculated from the μFTIR data (5 N/(year·capita)) proved to be approximately 332 times lower than that calculated from the μRaman dataset, although in line with previous studies employing μFTIR. By merging the MP length datasets from the two techniques, it could be shown that false negatives became prevalent in the μFTIR dataset already below 50 μm. Further, by fitting the overall frequency of the MP length ranges with a power function, it could be shown that μFTIR missed approximately 95.7% of the extrapolated MP population (1–1865.9 μm). Consequently, relying on only μFTIR may have led to underestimating the MP content of the investigated drinking water, as most of the 1–50 μm MP would have been missed.

## Highlights

• MP analysis with μFTIR and μRaman of drinking water samples from a waterworks.
• μFTIR experimental size limit 50 μm, nominal 6.6 μm.
• Accordance of the two methods for the retention of MPs >6.6 μm (∼80%).
• Human MP intake estimation from μFTIR ∼332 times lower than μRaman.

## Graphical abstract

**Figure:**
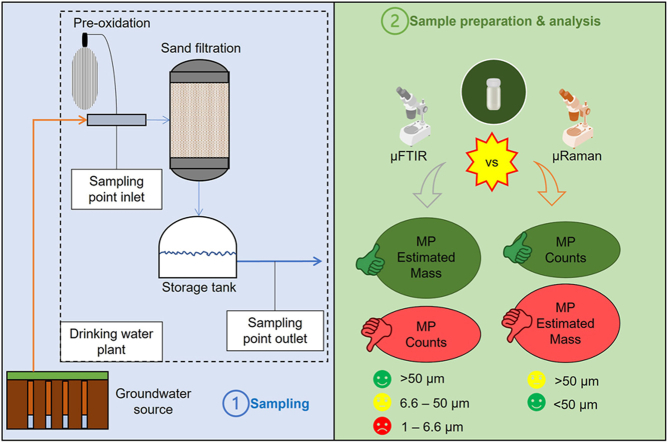

## 1 Introduction

Since the beginning of the century, when it was first demonstrated that microscopic plastic fragments and fibres accumulate in the marine environment [1], the study of microplastic pollution has evolved into a mature research field. Following the findings of Thompson et al. (2004) [1], a debate arose around the origin of these small plastic fragments and how they could be classified. Regarding the latter, a standard definition of the term “microplastic” (and its close relative “nanoplastic”) is still lacking, but plastic debris smaller than 1 μm is generally called *nanoplastics* (NPs), while *microplastics* (MPs) are commonly associated with particles between 5 mm and 1 μm [2]. Furthermore, plastic particles with a size between 1 and 5 mm are sometimes called “large microplastics” [3]. In the present paper, these conventions were also followed.

Regarding the origin of the plastics, intentionally produced microplastics, such as microbeads added to cosmetic and personal care products are one source of MPs [4], commonly called “primary microplastics”. A more common type is “secondary microplastics”, that is, MPs created by the fragmentation of everyday plastic objects [5]. The main parameter by which MPs are classified is the “size”, which is commonly defined as the major dimension of its 2D projection [6]. Size is important, as the toxic impact of MPs among others depends on particle size, where small MPs and NPs are believed to exert higher toxicity than larger ones [7]. For example, 1–10 μm poly-styrene (PS) MPs have been shown to cause necroptosis and inflammation in mice [8], and enhanced cellular uptake was observed in three human cell lines exposed to ∼1 μm PS and 400 nm poly-methylmethacrylate (PMMA) NPs [9]. Therefore, the accurate quantification of small sized MPs and NPs is an important topic due to the potential effects on human health as a consequence of daily exposure [10].

Human intake of MPs can occur *via* three major pathways, inhalation, dermal contact, and through the ingestion of food and beverages [11]. Also drinking water represents a source of MP intake by humans [12], with bottled water generally showing higher abundance than tap water [13]. Nonetheless, tap water can be an important contributor to human MP exposure. Tap water is produced by purifying a raw water source, and understanding MP occurrence before and after the treatment in the waterworks can provide useful insights into the efficiency of the facility in removing the MPs [14]. Typically, advanced drinking water plants employing a series of treatment steps show higher MP removal efficiency than simpler facilities [15].

For measuring MPs in drinking water, spectroscopic techniques have often been the method of choice [16], although no standardized protocols are yet agreed on for the analysis of drinking water [17]. Among the spectroscopic methods, Fourier-Transform InfraRed micro-spectroscopy (μFTIR) is by far the most widely used technique. It allows for analysing particles and fibres on an active area either on filters [18] or windows [19]. Today, many of these instruments are equipped with a Focal Plane Array (FPA) detector enabling them to take extensive chemical images in a relatively short time, even if complex analytes such as fibres are present [20].

Raman micro-spectroscopy (μRaman) is considered an alternative or complementary analytical technique to μFTIR. It is characterized by a finer spatial resolution in the XY plane than μFTIR [21]. Therefore, it is generally utilized for the smallest particle fraction down to around 0.4 μm, depending on the chosen analytical parameters. For particles above 10 μm, μFTIR is generally the preferred technique [21]. μRaman analysis is typically performed directly on the filters where the samples were previously deposited. Generally, a so-called “point and shoot” approach is followed: the instrument's laser is automatically driven to each particle, which is distinguished from the visible image's background by contrast [22]. Consequently, the analysis time of a μRaman investigation depends on the number of items to be analysed, the spectral acquisition time, and the number of spectral accumulations selected. Thus, μRaman is generally a more time-consuming method than μFTIR [23].

Up to now, there have been limited attempts to compare the outcome of these two techniques concerning the analysis of MPs in environmental samples. Marine water from the North Sea was analysed [24], and the results from μFTIR and μRaman were compared. The authors showed that the MP abundance in the same size ranges from μRaman data was higher than that estimated from μFTIR. The difference was due to the inherently better ability of μRaman to detect MPs below 10 μm, which were found to be the most abundant size fraction in the samples. More recently, atmosphere pollution from MPs has been studied. Gaston et al. (2020) [25] published a study where MPs from indoor and outdoor air were analysed with μFTIR and μRaman. In this case, the two techniques found different polymers in the samples for the size range 20–8961 μm: μFTIR data showed a prevalence of poly-styrene (PS), while μRaman indicated poly-vinyl chloride (PVC) as the most common plastic type. This discrepancy may be due to the different spectral identification methods used by the authors for the two techniques.

In the present work, we applied μFTIR to quantify the MP content of drinking water samples from a Danish waterworks and compared the result with that of μRaman, which we described in Maurizi et al. (2023) [26]. Although these two spectroscopic techniques have so far been the most widely employed for MP analysis in drinking water [27], a systematic experimental comparison between the two methods is still lacking for this environmental compartment. Hence, we believe that such a study is imperative to put the current state-of-the-art into perspective, in light of the upcoming requirement of the European regulation on MP monitoring in drinking water [28].

## 2 Materials and methods

### 2.1 Sampling of drinking water and field blanks

The waterwork structure and sampling process were described in Maurizi et al. (2023) [26]. In short, the investigated waterworks produces around 500,000 m^3^/year of drinking water from groundwater, utilizing a simple treatment process consisting only of oxidation and rapid sand filtration. The groundwater is extracted from a limestone aquifer at a depth of 72–92 m. A network of pumps and pipes transfers the raw water from the wells to the waterworks. After the treatment, the water is retained in two tanks with a storage capacity of 110 m^3^ each, corresponding to a retention time of approximately 2–3 h during the daytime. From here, the water is pumped into the distribution network.

The sampling campaign took place between the 28th of September and the 1st of October, 2021. Both the waterworks' inlet and outlet were sampled for five consecutive days, from 9 a.m. to 2 p.m. with two custom-made sampling devices. Each sampling device consisted of four flow lines hosting one or two filter holders and terminating with a flowmeter to quantify the sampled volume. The inlet of the devices was connected to the sampling points by a flexible steel tube. Approximately 1 m^3^ of drinking water was filtered on each of the filters (1 μm sintered steel filters (Mesh Masters, The Netherlands)) along the first three flow lines of the two sampling devices (3 L/min). Inlet and outlet were sampled in parallel, resulting therefore in a total of 5 inlet and 5 outlet samples collected in triplicates (n = 30).

For the field blanks, the fourth flow line of the two sampling devices was equipped with two filter holders in series. The first one hosted a muffled 0.7 μm glass fibre membrane (Th. Geyer GmbH, Germany), whilst the second one held a 1 μm sintered steel filter. Approximately 20 L of drinking water were pre-filtered through the glass fibre membrane onto the sintered steel filter for each field blank. Two field blanks were sampled in parallel on each day, thus obtaining 10 replicates in total. The volume sampled for the field blanks was lower than that of the drinking water samples since blank contamination mainly occurs during sample preparation at the laboratory and is not related to the amount of sampled water. The 1 μm sintered steel filters were stored in Petri dishes before sample preparation. Further information on the waterworks and the sampling is available in the Supplementary Information.

### 2.2 Contamination prevention

All chemicals were filtered through muffled glass fibre membranes to remove any particles and fibres above 0.7 μm. The glassware was muffled at 500 °C for 4 h, and metallic labware was thoroughly rinsed with particle-free water before use. Moreover, the filters were stored in muffled Petri dishes inside a laminar flow bench until treatment. Pure cotton lab coats were worn during the entire sample preparation, and the usage of plastic gloves was avoided during sample preparation. All sample preparation was done in a laminar flow bench, which was regularly cleaned with 50% EtOH.

### 2.3 Sample preparation and deposition

The sample preparation protocol for the μRaman analysis was presented in Maurizi et al. (2023) [26] (see also Supplementary Information, section 2 and Fig. S3). In short: after the sampling, the 1 μm sintered steel filters were incubated in 5% SDS (Sodium Dodecylsulphate, Th. Geyer GmbH, Germany) at 50 °C for 24 h, and, after 5 min of ultrasonication, the particle-enriched SDS mixture was filtered through the same filter with a vacuum filtering system. After a further round of ultrasonication in 50% EtOH (EtOH for HPLC, Th. Geyer GmbH, Germany) and vacuum filtration, the particles on each filter were recovered in 50% EtOH, and the ethanolic mixture was poured into a 10 mL vial. Finally, the mixture was dried at 55 °C under gentle nitrogen flux in a water bath (TurboVap Biotage, Sweden) and reconstituted with 1000 μL of ∼99.5% EtOH. The μRaman analysis was performed on the active area of each sample. The active area was obtained by depositing 25 μL per sample on 10 × 10 mm Silicon substrates through a metallic funnel with a diameter of 2 mm.

For the μFTIR analysis, the same samples were dried under gentle nitrogen flow in a water bath at 55 °C and reconstituted with 2000 μL of 50% EtOH. The deposition was performed by means of compression cells onto 13 mm diameter Zinc Selenide (ZnSe, Crystran Ltd., UK) windows of 2 mm thickness with a manual pipette equipped with 200 μL glass tips (Brand GmbH, Germany). 1000 μL were deposed for the drinking water samples, and 600 μL for the field blanks. The samples were dried at 55 °C on a heating plate (W10 VWR, Denmark) overnight, which produced an active area of approx. 10 mm in diameter. Sample reconstitution, deposition, and analysis took place in March–September 2022.

### 2.4 μFTIR analysis and spectral recognition

Analysis was conducted with a Focal Plane Array (FPA) – μFTIR. A Cary 620 FTIR microscope coupled with a Cary 670 IR spectroscope (Agilent Technologies, USA) was used to scan the active area of the enriched ZnSe transmission windows. The microscope was equipped with a 25 × Cassegrain objective, producing 3.3 μm pixel resolution on a 128 × 128 mercury cadmium telluride (MCT) FPA detector. The detector was cooled down to the operative temperature of 80K with liquid nitrogen continuously pumped into the FPA dewar from a holding tank (Norhof, The Netherlands). All scans were carried out in transmission mode with a spectral range of 3750–850 cm^−1^ at 8 cm^−1^ resolution applying 30 co-added scans in transmission mode. A background tile was collected before each sample's scan, using the same parameters, but co-adding 120 scans instead of 30. A chemical image of the sample's active area was obtained, where each pixel contained an IR background-corrected spectrum.

The μFTIR chemical images were analysed with the software siMPle [29] v. 1.3.1β. It can automatically detect the particles in a μFTIR spectral image, determine their morphological parameters, and estimate their volume and mass. Specifically, the volume of a particle was estimated from its 2-dimensional projection by first calculating its equivalent ellipse where the major diameter equals its longest Feret diameter, then calculating an equivalent ellipsoid where the third diameter equals 0.6 times the second diameter of the equivalent ellipse. The mass was then found by multiplying the volume of the ellipsoid by the density of the identified polymer ([6,30]).

Each pixel containing an IR spectrum was compared with the built-in software's library, which led to an estimation of the Pearson correlation coefficient. Both the original spectrum and the first derivative were compared with the library's references (Supplementary Information, section 5 for examples of experimental μFTIR spectra) after an automatic removal of the CO_2_ peaks. Moreover, spectral matches from the software were manually checked to further assess their reliability.

Considering the filters' pore size (1 μm), the nominal pixel size of the μFTIR at 25 × magnification (3.3 μm), and the observation that objects represented by only one pixel on the FPA image sometimes were false positives, it was decided to include only objects occupying at least 3 pixels, corresponding to a triangular shape (i.e. particles of a minimum length of 6.6 μm) [30]. Cellulose and protein particles could also be successfully identified in the samples as done with the MPs, and they represented the non-plastic fraction in the MPs/non-plastic ratio quantification.

The μRaman analysis of the blanks and drinking water samples was conducted as reported in Maurizi et al. (2023) [26]. Briefly: a visible montage of the sample's active area was taken at 50 × , and the spectral acquisition was automatically performed at 100 × with a 532 nm laser (power 50 mW, grating 1200 ll/mm) onto each particle above 1 μm in the visible picture. The spectral recognition was conducted with the commercial library associated with the software of the μRaman system, and the particle mass was estimated as done for the μFTIR data.

### 2.5 Post-processing of the μFTIR data

For each drinking water sample replicate, the MP counts and estimated mass was calculated as the sum of the counts or estimated mass of each identified polymer. The values of MP abundance were corrected by subtracting the corresponding blank mean. Then, the values for the three replicates were averaged and normalized to 1 m^3^, thus leading to the MP counts in N/m^3^ and estimated MP mass in μg/m^3^.

The waterworks' removal efficiency *R* was calculated as the cumulated efficiency over the five investigated days. Hence the sum of the MP counts or mass abundance at the inlet ([*MPs*_*inlet*_]) was compared with that of the outlet ([*MPs*_*outlet*_]) according to equation (1):

(1) $$R=-(\left(\left[{\mathrm{MPs}}_{\mathrm{outlet}}\right]–\;\left[{\mathrm{MPs}}_{\mathrm{inlet}}\right])/\left[{\mathrm{MPs}}_{\mathrm{inlet}}\right]\right)\cdot 100\%\;$$

Uncertainty was calculated by selecting a coverage factor *k* = 2 (*P* = *95%*) and considering only the A-type (statistical) contribution. The statistical tests and non-linear regression of the MP length frequency were performed with Rstudio v. 2022.02.1 + 461. Normality was assessed with the Shapiro-Wilk test. ANOVA was conducted for significance assessment (α = 0.05) and Tukey-test for pair-wise factor comparison. The samples were labelled as Dxy, where D means “Day”, x is the sampling day (1–5), and y is either i or o (i = inlet, o = outlet).

The MP quantification from the μRaman analysis was described in Maurizi et al. (2023) [26]. Briefly: for each polymer in the replicates, the MP counts and estimated mass were corrected by subtracting the blank mean, and the value obtained was considered only if above the corresponding LOQ. The MP counts and estimated mass for each replicate were obtained as the sum of the polymers above the LOQ, and then the three replicates were averaged. Equation (1) was finally applied to calculate the MP removal efficiency over the investigated period and also according to the MP size ranges 1–10 μm, 10–20 μm, 20–50 μm, and 50+ μm.

## 3 Results and discussion

### 3.1 Microplastic morphological analysis

**Fig. 1:**
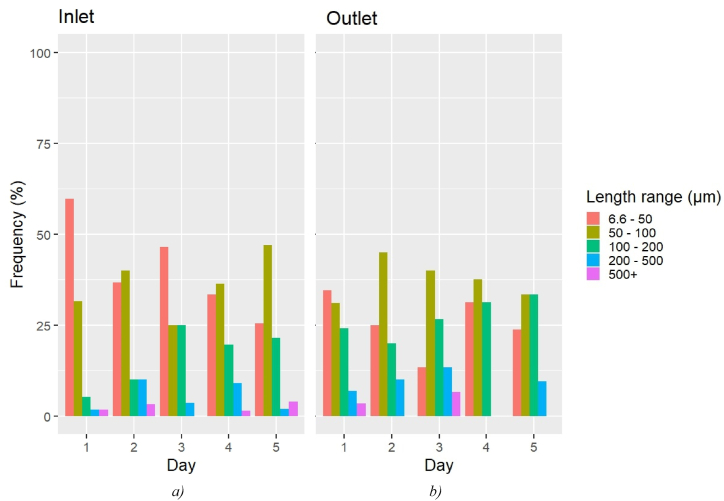
Barplots of the MP length range frequency over Days 1–5 at the waterworks' a) inlet and b) outlet. Values not corrected for blank contamination (μFTIR analysis).

The morphological data from the μFTIR analysis were not blank-corrected. The MPs were grouped into five length ranges (6.6–50 μm, 50–100 μm, 100–200 μm, 200–500 μm, and 500+ μm), and their frequency was calculated for each investigated day for both inlet and outlet (Fig. 1).

In the inlet samples, the majority of MPs belonged to the 6.6–50 μm length range (mean 40.1%), followed by those sized between 50 and 100 μm (mean 36.6%). The remaining three length ranges accounted for approximately 23% of the entire inlet MP population. In the outlet samples, the 50–100 μm length range was the most populated (mean 36.6%), whilst the 6.6–50 μm and the 100–200 μm length ranges were equally represented (mean 26.7%). The MPs above 200 μm accounted for approximately 10% (p < 0.05).

According to Cabernard et al. (2018) [24] and Kooi and Koelmans (2019) [31], the MP size frequency increases with decreasing MP size according to a power law. However, Fig. 1 suggests that this was not the case for the MP size data from the μFTIR analysis reported in this work, where the 6.6–50 μm MP frequency was generally comparable with that of 50–100 μm MPs. Hence μFTIR may have missed some of the MPs below 50 μm during the analysis, thus leading to an MP size frequency differing from what was expected (see also section 3.3). Dalmau-Soler et al. (2021) [32] and Jung et al. (2022) [33], who investigated the MP occurrence down to 20 μm in drinking water plants in respectively Korea and Spain with μFTIR, also reported that the frequency of >200 μm MP decreased after the water treatment.

The largest analysed MP was 1865.9 μm long and the median length was highest in the outlet samples (65.3 *versus* 60.5 μm at the inlet). The smallest detected MP was 13.3 μm long, approximately twice the theoretical size limit considered acceptable with the employed FPA detector (see section 2.4). This may likely be due to the recognition thresholds chosen in the siMPle software upon the spectral matching, which may have caused the dismissal of potential MPs due to a low hit quality. The spectral quality of an IR transmission analysis is strongly affected by the thickness of the sample [34], and it rapidly deteriorates if thin samples, like MPs <20 μm, are analysed. Therefore, setting the quality thresholds was subject to a compromise between reducing the false positives and recovering as many MPs as possible from the IR maps of the samples.

The MP shape was investigated by defining those with a length/width ratio greater or equal to 3 as fibres, otherwise as fragments [35]. For both morphotypes, the inlet samples presented higher counts of items than the outlet samples except for D1, where six fibres were found in the inlet samples and 8 in those of the outlet. Overall, the fragments accounted for 79.3% of the analysed MPs, the rest being fibres.

These findings were strongly different from what was found in the μRaman investigation [26], where the MPs longer than 50 μm represented the least populated fraction, and the largest item was 204.6 μm long (median length inlet and outlet 2.4 μm). In addition, the fibres represented less than 2% of the total MP population in the μRaman dataset, which suggested the fibre-like morphotype to be increasingly rare as smaller size ranges are considered. This observation is supported by Feld et al. (2021) [36], who employed μFTIR to analyse MPs in Danish drinking water above 100 μm and found that the majority had a fibre shape.

### 3.2 Microplastic quantification with μFTIR

#### 3.2.1 Microplastics in the field blanks

Despite great care being exercised when preparing the samples, MPs were found in the field blanks. The mean counts estimate was 13.4 ± 12.0 N/m^3^, with the following polymeric frequency: PVC 31.9%, poly-ester (PET) 25.5%, poly-propylene (PP) 25.5%, poly-vinyl dichloride (PVDC) 12.8%, and alkyd 4.2%. The mean mass estimate was 2.0 ± 2.3 μg/m^3^, with the following polymeric composition: PET 60.9%, PVC 21.2%, PP 13.5%, alkyd 3.2%, and PVDC 1.2%. All inlet samples presented a mean MP abundance above the blank mean, whilst the MP abundance of the outlet samples was lower (with the exception of D1 and D3 for the estimated mass). Since in the drinking water samples there were also different polymers from those in the blanks, the mean MP counts of the blanks have to be considered indicative.

#### 3.2.2 Microplastic abundance in the drinking water samples

**Fig. 2:**
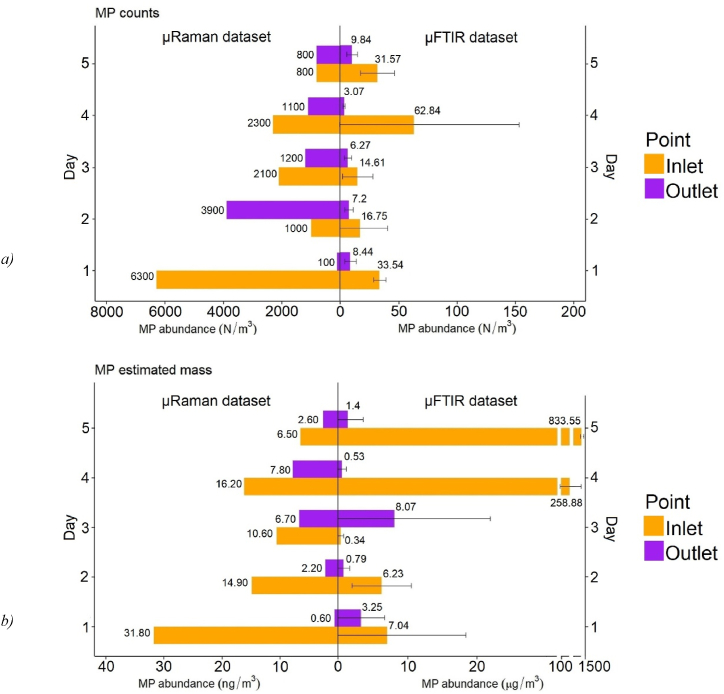
MP a) counts and b) estimated mass over the investigated period according to the μFTIR and μRaman analysis. Notice the unit of the x-axes.

Combining the data from μFTIR and Raman, Fig. 2 shows the MP abundance according to the counts and mass estimates for both sampling locations on each investigated day. The source of the error for the μFTIR data might come from a true variability between the triplicates, or it might be an artifact of the deposition procedure, as only half of the sample extract was deposited and analysed. That is, the MPs might have been inhomogeneously distributed inside the vials while the deposition took place. For the MP mass estimate, the usage of the ellipsoid model is an additional source of uncertainty, as it cannot fully cover the variety of MP morphotypes. Furthermore, the data in Fig. 2 show the sum of all polymers identified, hence the error also depends on the variation associated with the single polymers.

The outcome of the μFTIR analysis was not in line with that of the μRaman study. According to the μFTIR data, none of the five days had MP counts at the inlet higher than that at the outlet. Consequently, the ANOVA and Tukey test showed a significant difference (p < 0.05) between “inlet” and “outlet”, which meant the waterworks removed MPs. The μRaman investigation on the other hand showed that the MP counts at the outlet on D2 were higher (3900 N/m^3^) than that at the inlet (1000 N/m^3^). The MP mass estimate provided by μFTIR was approximately 23 times higher at the outlet (8.07 ± 16.05 μg/m^3^) than at the inlet (0.34 ± 0.49 μg/m^3^) on D3, whilst on the remaining four days it was always lower at the outlet. The single event that occurred on D3 did not influence the outlet values on D4 and D5, which showed a strong retainment by the waterworks. Scrutinizing the data, it became clear that a few but large-sized MPs were released on D3, which accounted for the mass at the outlet being larger than at the inlet. On average, the μFTIR analysis yielded 31.86 ± 17.17 N/m^3^ at the inlet and 6.96 ± 2.27 N/m^3^ at the outlet, whilst the mean MP mass estimate was 221.21 ± 320.04 μg/m^3^ at the inlet and 2.81 ± 2.78 μg/m^3^ at the outlet (Fig. 2). Past studies employing μFTIR for the MP quantification of drinking water obtained diverse results, primarily due to the different size limits set during the investigation and the sampling protocols adopted. Mintenig et al. (2019) [37] analysed groundwater-derived drinking water in Germany and found negligible concentrations of MPs (<1 N/m^3^) sized above 20 μm (300–2500 L of drinking water per sample). Similar results were obtained in Spain for tap water analysed with μFTIR for MPs above 50 μm [38] (60 L of drinking water per sample). Mukotaka et al. (2021) [39] published a comprehensive study targeting MPs above 20 μm with μFTIR in tap water from Japan, the USA, France, Germany, and Finland and found abundance values between 1.9 and 225 N/L (500 mL of drinking water per sample). Hence higher MP abundance was found with μFTIR when smaller volumes of drinking water were sampled.

The mean MP counts provided by the μRaman analysis [26] were markedly higher (inlet: 2500 ± 2000 N/m^3^, outlet: 1400 ± 1300 N/m^3^), whilst the mean MP mass was approximately 10^−3^ times lower (inlet: 16.0 ± 8.5 ng/m^3^, outlet: 4.0 ± 2.7 ng/m^3^). Cabernard et al. (2018) [24] also found that μRaman led to far higher MP counts than μFTIR (39–2621 N/m^3^ and 22–228 N/m^3^, respectively) when applied to the MP analysis of marine water. The authors addressed the higher degree of automatization of μRaman as the main reason why this method proved to be more efficient in recognizing the 10–500 μm MP. In the case of this work, the μFTIR system performed automatized FPA mapping, so the bias was minimal, and no manual selection of particles was necessary. Hence the discrepancy between μFTIR and μRaman regarding the MP abundance in the analysed drinking water samples should mainly be associated with the different spatial resolution of the two methods, which for μFTIR seems larger than expected (sections 3, 3.1.3). Comparing the outcomes of the two methods, it is evident that the usage of only μFTIR would have led to a ∼150 times underestimation of the MP counts abundance in the analysed drinking water. Accordingly, μRaman was shown to provide higher MP abundance values in drinking water, in accordance with studies conducted in the Czech Republic [40] (up to 3605 N/L in raw water and 628 N/L in treated water), China [41] (440 N/L on average in tap water from 38 cities), and Iran [42] (up to 2808 N/L in raw water and 1401 N/L in treated water).

Gaston et al. (2020) [25] also reported that μFTIR underestimated the MP counts abundance of air samples if compared with μRaman by approx. 10 times and by manually selecting the particles to be analysed. Quantification of NPs in actual waterworks operating under real conditions has been a neglected topic so far. To the best of the authors' knowledge, only Li et al. (2022) [43] quantified NPs in tap water. They used Pyr-GC/MS by sequentially isolating nanoparticles down to 20 nm with a cascade filtration approach.

#### 3.2.3 Removal efficiency of the waterworks

The MP counts removal efficiency calculated from the μFTIR analysis was higher (78.1 ± 49.7%) than that calculated from the μRaman dataset (43.2 ± 45.9%). Moreover, the counts removal efficiency as per the μFTIR exhibited an increasing tendency over the investigated five days, contrary to the decreasing tendency shown by μRaman. Nonetheless, the MP counts removal efficiency provided by μFTIR was in line not only with previous studies from the literature ([44,45]) but also with the value obtained for the 6.6+ μm MPs from the μRaman dataset (81.3%). However, the μRaman analysis also showed that the removal efficiency of the 1–6.6 μm MP was approx. 40%, a finding not provided by μFTIR (due to differences in nominal size limits) and which could explain the overall lower value of MP counts retainment as per μRaman.

A similar picture emerged from the MP mass removal efficiency. In this case, the μFTIR analysis provided a mean value of 98.7 ± 11.1% *versus* 75.1 ± 28.2% from μRaman. Although the MPs sized 1–6.6 μm approx. represented the majority in terms of MP counts in the μRaman dataset, they did not account for a large mass altogether, which was in the order of ng/m^3^. On the contrary, the MPs identified by μFTIR, being larger, held a mass in the order of μg/m^3^. Hence the influence of the smaller MPs on the mass balance was not decisive and close values of MP mass removal efficiency could be obtained with the two techniques. Overall, these findings indicated that larger MPs could be more efficiently retained than smaller ones, which is in accordance with previous studies on deep bed filtration in granular media ([[46], [47], [48], [49]]).

μFTIR also allowed for the identification of non-plastic particles, namely cellulose and proteic polymers [25], whilst during the μRaman analysis the non-plastic particles could not be identified [26]. Overall, the MPs/non-plastic ratio provided by μFTIR was generally higher (inlet 30.0–468.7%, outlet 7.6–79.5%) than that of μRaman (inlet 0.9–29.0%, outlet 0.3–21.5%). Therefore, μRaman may have been less efficient in detecting the MPs (i.e. false negatives showing a not recognizable Raman spectrum due to fluorescence and low scattering), which led to a smaller fraction of the MPs in the active area being identified. In this regard, the MPs/non-plastic ratio may also be considered an efficiency parameter of the analytical device employed. However, it should be considered that the quality thresholds set in the software siMPle also played a role in determining the MP counts calculated in the drinking water samples as per the μFTIR analysis, as well as the spectral hit quality criteria chosen in the μRaman study.

#### 3.2.4 MP composition of the drinking water samples

**Fig. 3:**
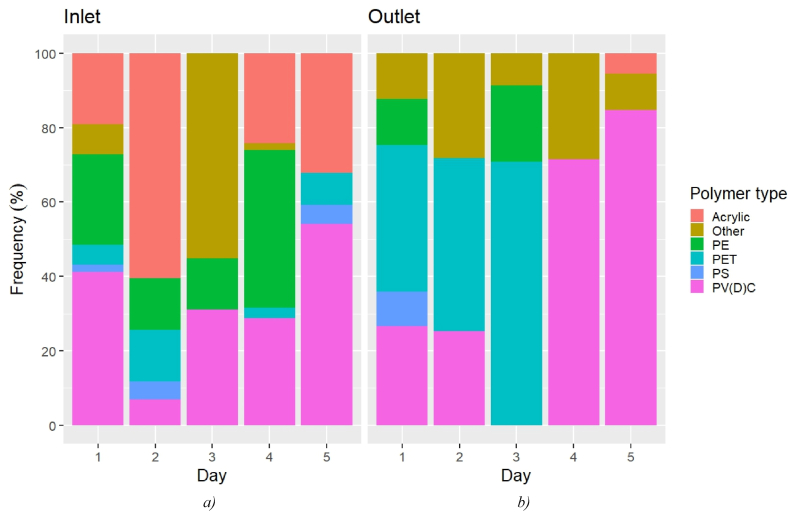
MP frequency at the a) inlet and b) outlet over the investigated period according to the μFTIR analysis (counts).

**Fig. 4:**
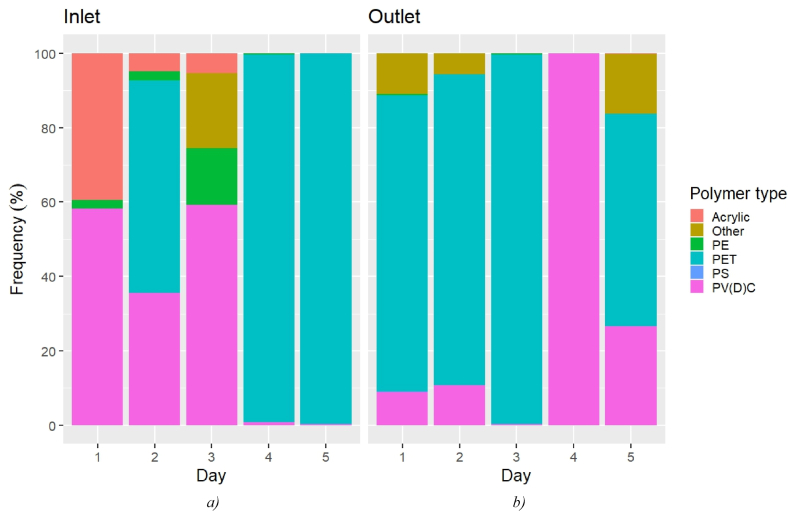
MP frequency at the a) inlet and b) outlet over the investigated period according to the μFTIR analysis (estimated mass).

The polymer frequency (as determined by μFTIR) in the outlet samples was characterized by an increase in PET in both the MP counts and estimated mass, as shown in Fig. 3, Fig. 4. According to the counts estimate (Fig. 3), the most abundant polymeric category at the inlet was PV(D)C (7.0%–54.0%), followed by acrylic (0–60.4%) and PE (0–42.5%). At the outlet, PV(D)C was again the most abundant plastic (0–84.7%), but PET immediately followed (0.0–70.8%). No enrichment in PA at the outlet could be seen in this case, contrary to what was shown by μRaman (mean inlet 39%, mean outlet 78%). This was not surprising, considering that the majority of the PA particles were sized 1–5 μm, so they could not be analysed by μFTIR.

The mass estimate showed a different picture (Fig. 4). At the inlet, PET was indeed the most common polymer (mean 97.9%), followed by PV(D)C (mean 1.45%). At the outlet, PET accounted for 88.7% on average (0–99.2%), resulting in the most abundant polymer of the mass estimate. Therefore, the higher MP mass abundance at the outlet on D3 as per the μFTIR analysis may have been caused by a temporary leaking of PET particles sized above 6.6 μm. On the other hand, PA (mean inlet 37%, mean outlet 34%) and poly-acrylics (mean inlet 19%, mean outlet 65%) were the most frequent polymers in the mass estimate provided by μRaman. Overall, the significance test could not associate any of the polymer groups identified by μFTIR with a contribution in terms of a *persistent* release of MPs during the treatment. On the contrary, a significative and systematic presence of PA particles at the outlet was identified by μRaman.

### 3.3 Overall comparison between μFTIR and μRaman

**Fig. 5:**
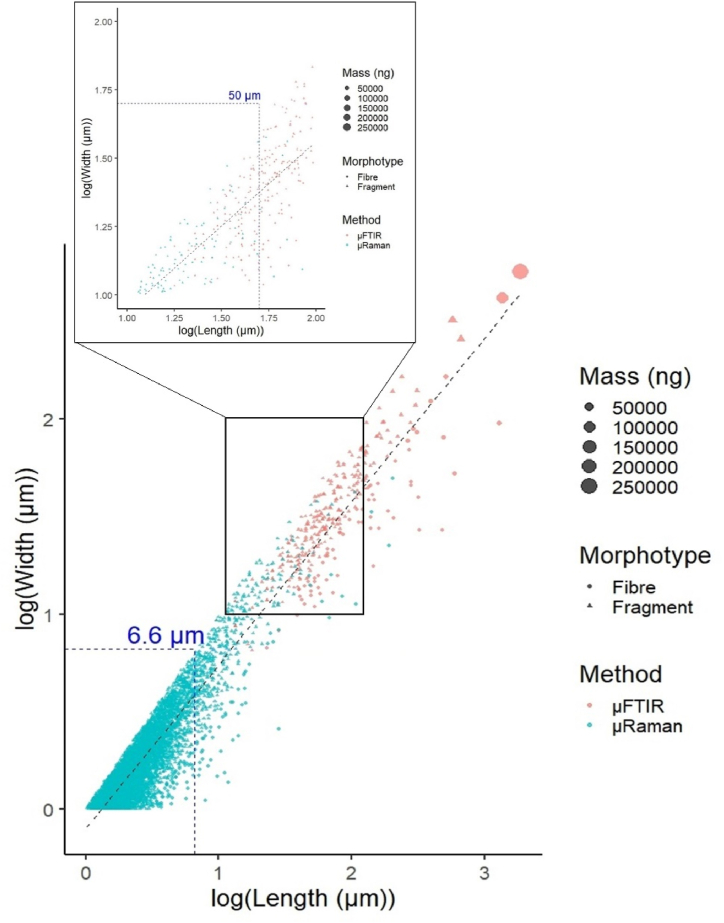
Width vs length graph for all the analysed MPs in the two datasets (the axes scale is in log_10_). On the left: detail of the small-sized MP distribution. Values not corrected for blank contamination.

Fig. 5 graphically emphasizes the two distinct “MP size domains” of μFTIR and μRaman, by plotting the width *vs* length of all the analysed MPs in Maurizi et al. (2023) [26] and the current study in a log_10_ graph. As can be seen, between 1 and 2 of the log (Length) (10–100 μm), μFTIR started missing the smaller MPs, and data from μRaman became prevalent. Hence the experimental size quantification limit for μFTIR (at the applied analysis conditions) proved to be approx. in the centre of this range (50 μm), well above the nominal one (6.6 μm at 25 × ). Accordingly, in section 3.1 it was observed that the frequency of 6.6–50 μm MP was lower than expected from the literature. This does not mean that μFTIR cannot analyse MPs below 50 μm (the smallest MP identified in the present study by μFTIR was indeed 13.3 μm long), but rather, that the false negatives risk overcoming the true positives. Therefore, the μFTIR data associated with the 6.6–50 μm range should be considered indicative not only in the present study, but also in the previous works based on μFTIR reported in the literature. Consequently, μRaman should ideally be employed to efficiently target MPs below 50 μm, whilst μFTIR proved more suited for MPs above 50 μm. However, the final performance of the methods might also be influenced by the deposition protocol adopted for the samples prior to the analysis (i.e. different percentages of deposited volume) as well as the spectral data post-processing, which was performed with two distinct software. Hence the choice of either method for MP analysis should always be contextualised in the reality of the analytical process, thus reaching a compromise between accuracy, analysis time, and demands from the state-of-the-art.

**Fig. 6:**
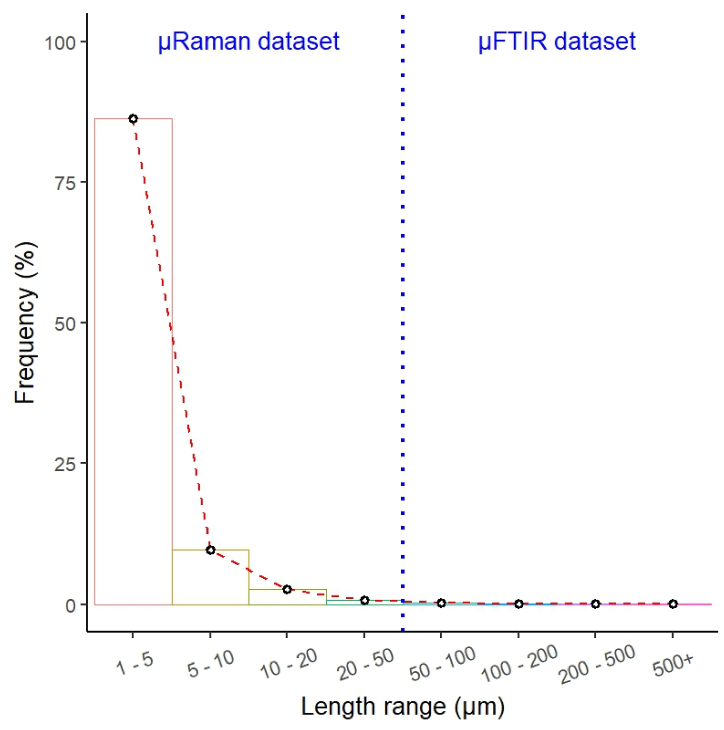
Frequency distribution of 1–1865.9 μm MPs in the analysed drinking water samples. Values not corrected for blank contamination. The blue dashed line divides the length ranges according to the relevant spectroscopic dataset.

The MP length datasets from the inlet and outlet provided by the two techniques were merged to extrapolate the MP population from 1 (size limit of μRaman [26]) to 1865.9 μm (largest MP identified by μFTIR in the present study) and quantify the percentage of MPs missed by μFTIR. For the 1–50 μm interval (1–5, 5–10, 10–20, and 20–50 μm), the μRaman dataset was employed, while the μFTIR data was used for the 50+ μm interval (50–100, 100–200, 200–500, and 500+ μm). The outcome of this investigation is illustrated in Fig. 6.

86.0% of the identified MPs had a length between 1 and 5 μm. The other length categories accounted increasingly less with increasing MP size (5–10 μm 9.7%, 10–20 μm 2.7%, 20–500+ μm 1.3%).

Therefore, the MP length distribution was skewed toward the left (i.e. the smallest values of MP length), and μFTIR missed approx. 95.7% of the extrapolated MP population. Specifically, the frequency values could be fitted against the overall MP length range of 1–1865.9 μm according to a power function (2):

(2) $$\text{Frequency}\;\left(\%\right)=133.88\cdot {\text{Length}}^{-3.933}\;\left(R^{2}=0.99\right)$$

Equation (2) was obtained by a non-linear regression using the package nlraa of R and presented in the same form as the theorical power function developed by Kooi and Koelmans [31] for the MP size distribution from various environmental matrices.

μFTIR and μRaman clearly performed differently during the analysis of the drinking water samples, omitting the fact that μFTIR could not analyse the MPs between 1 and 6.6 μm. The first reason for this outcome is associated with the finer spatial resolution of the μRaman, which is usually addressed as the main advantage of the technique over μFTIR [50]. Therefore, the μRaman dataset was mostly populated by the smaller particles since they were the most abundant in the samples. The second reason may be due to the different sample deposition methods employed: for the μRaman, 25 μL over 1000 μL were deposited (2.5% of the total volume), while for μFTIR 1000 μL over 2000 μL (50% of the total volume). This meant the probability to deposit larger particles was higher in the μFTIR protocol, given their scarce frequency in comparison with the smaller size fractions. Since the stage is moved according to the coordinates of each particle to be excited with a laser of choice, the analysis time with μRaman can sometimes be prohibitive and semi-quantitative strategies may have to be adopted (e.g. choice of sub-areas and quantification of the total counts of particles afterward). FPA-μFTIR, on the other hand, was used by taking advantage of its ability to map quite extended surfaces relatively quickly, despite its lower spatial resolution. The third reason, as already mentioned, may be represented by the settings employed in the software siMPle for the μFTIR data post-processing, and, in particular, the decision to ignore MPs smaller than 3 pixels on the chemical map. In general, relying only on μFTIR could have led to a severe underestimation of the MP abundance, or alternatively to an acceptance of a higher ratio of false positives, as discussed in section 3.2 and by Cabernard et al. (2018) [24].

**Fig. 7:**
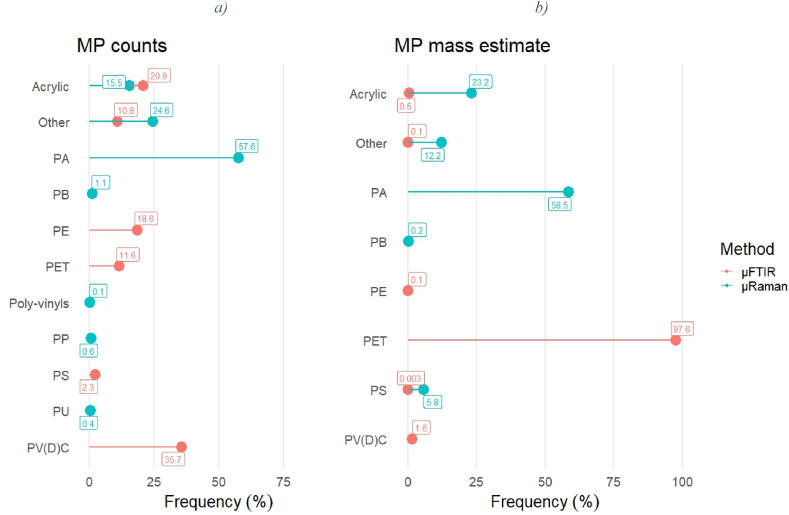
“Frequency of appearance” of the polymers identified by μFTIR and μRaman according to the MP a) counts and b) estimated mass.

The “frequency of appearance” of the identified polymer groups in the μFTIR and μRaman datasets also proved to be different (Fig. 7). Since blank correction was performed separately for the MP counts and mass estimation, some plastic groups present in the counts investigation could not lead to values higher than the blanks’ mean, so they were excluded. μRaman could identify more plastic groups than μFTIR (the “Other” category represents 9 distinct minor polymers in the μRaman dataset and 8 in that of μFTIR). In the plastic groups recognized by both methods, the frequencies did not prove to be comparable for PA, acrylic, and PP. In the case of PA, most of the particles were sized 1–5 μm, so they could exclusively be analysed with the μRaman. The opposite applied to acrylic, which was less abundant in the μRaman dataset because of its lower counts when compared to the smaller MPs. Most of the plastic mass in the μFTIR dataset was represented by PET which could not be quantified in the μRaman analysis.

### 3.4 Estimations of human MP intake from drinking water: a method-related issue

By adopting the same approach outlined in the μRaman study, it was possible to estimate the MP intake for an average drinking water consumer also from the μFTIR data. Specifically, the 6.6–150 μm MP, which are more likely to translocate to the gut epithelium according to the EFSA [51], represented 90.9% of the analysed MPs in the outlet samples. Therefore, assuming a mean water consumption of 2 L/(day·capita) [52] with an MP counts equal to the waterworks' outlet mean (6.96 N/m^3^), the final result was (3):

(3) $$\text{Intake}=6.96\;N/m^{3}\times 2\;L/\left(\text{day}\cdot \text{capita}\right)\times 90.9\%\times 365\;\text{day}/\text{year}\approx 5\;N/\left(\text{year}\cdot \text{capita}\right)$$

Corresponding to 1.86 μg/(year·capita) if the mean outlet MP mass (2.81 μg/m^3^) is used in equation (3). The outcome was consistent with the findings of Kirstein et al. (2021) [30] and Dronjak et al. (2022) [53] (27 N/(year·capita)), who employed μFTIR down to respectively 6.6 and 20 μm. However, it was also decidedly lower (∼332 times) than the estimation provided by the μRaman analysis (1533 N/(year·capita)). The strongly different estimations for the human MP intake from drinking water obtained with μFTIR and μRaman clearly represent a method-related issue, as also discussed in sections 3, 3.2.3 for the MP quantification and morphology. This is a point to be considered especially by those regulatory bodies involved in the monitoring of MPs in drinking water, such as the European Union (EU) [28], the State of California [54], and the United Nations (UN) [55]. MP bio-toxicity is a complex phenomenon depending not only on the number of MPs taken in by an organism in a certain period of time but also involving particle size, shape, and polymer type [56]. Specifically for higher organisms, MPs smaller than 5–10 μm can interfere with intestinal homeostasis and behavior [57,58]. Further, MP fibres are believed to share with other pollutants (e.g. asbestos) an increased bio-reactivity thanks to their elongated shape [59]. The polymer type may also play a role in MP toxicity due to the leaking of unreacted monomers and additives (e.g. PCB plasticizers) after entering the organism [60]. This multi-faceted character of MPs as potential toxic agents renders it extremely difficult to address the effect of MP intake in humans as a whole [61]. Hence future regulations on MPs should be based on a comprehensive background taking into account the pros and cons of the analytical methods usually employed for MP analysis, thus clarifying the perimeter of applicability of the legal requirements.

## 4 Conclusion

Mainly because of their spatial resolution limit, μFTIR and μRaman provided different outcomes for the MP amount in the investigated drinking water samples, and, consequently, on the MP occurrence between the waterworks' inlet and outlet. By merging the two datasets, it was possible to demonstrate that the experimental size quantification limit for μFTIR was approx. 50 μm, in contrast to the nominal one of 6.6 μm at 25 ×. Below 50 μm, the rate of false positives associated with μFTIR makes μRaman the method of choice for MP analysis in drinking water. Further, the MP frequency in the range 1–1865.9 μm could be fitted *vs.* the MP length with a power function, showing that the 1–10 μm MP represented the majority of the MP population in the drinking water samples. These outcomes seem to suggest that previous investigations relying only on μFTIR could have underestimated the MP abundance in drinking water.

Overall, μFTIR provided lower (∼150 times) values of MP counts in the investigated drinking water samples than μRaman. The estimated MP mass, on the other hand, was approximately 10^3^ times higher in the μFTIR dataset due to the larger size characterizing the identified MPs. Remarkably, the counts removal efficiency for the MPs above 6.6 μm calculated with μFTIR data proved to be close to that of μRaman (∼80%). Hence the investigated waterworks poorly retained the MPs sized approx. 1–10 μm and proved to be highly efficient for the larger ones. The estimation of the human MP intake provided by μFTIR as MP counts was also decidedly lower (∼332 times) than that obtained from the μRaman analysis. This study is an important step forward in understanding the cons and the pros characterising μFTIR and μRaman as premiere techniques for the MP analysis of drinking water, as well as their complementarity and pivotal role in the MP science field. It also contributes to addressing the lack of standardization affecting the MP science field, which still hinders the routine monitoring of microplastic levels in drinking water, and puts into perspective the outcomes of past studies.

## Funding

This work has received funding from the European Union's Framework Programme for Research and Innovation 10.13039/501100007601Horizon 2020 under the Marie Skłodowska-Curie Grant Agreement MONPLAS No. 860775. The authors declare that they have no conflict of interest.

## Author contribution statement

Luca Maurizi conceived and designed the experiments; performed the experiments; analysed and interpreted the data; contributed reagents, materials, analysis tools or data; wrote the paper. Lucian Iordachescu performed the experiments; analysed and interpreted the data; contributed reagents, materials, analysis tools or data; wrote the paper. Inga V. Kirstein conceived and designed the experiments; analysed and interpreted the data; wrote the paper. Asbjørn H. Nielsen analysed and interpreted the data; wrote the paper. Jes Vollertsen analysed and interpreted the data; wrote the paper.

## Data availability statement

Data will be made available on request.

## Declaration of competing interest

The authors declare the following financial interests/personal relationships which may be considered as potential competing interests: Luca Maurizi reports financial support was provided by European Commission.
