# Supplementary material for: It matters how we measure - Quantification of microplastics in drinking water by μFTIR and μRaman
Source: Heliyon. 2023 Sep 13;9(9):e20119. doi: 10.1016/j.heliyon.2023.e20119 (PMC10559862; doi:10.1016/j.heliyon.2023.e20119)
Supplement: Multimedia component 1 [file mmc1.docx]

It matters how we measure - Quantification of microplastics in drinking water by µFTIR and µRaman

L. Maurizi, L. Iordachescu, I. V. Kirstein, A. H. Nielsen, J. Vollertsen

**Supplementary Information**

# Waterworks and sampling apparatus

Figure S1 and S2 respectively represent the investigated waterworks and the device employed for the drinking water sampling (Maurizi *et al.* 2023).


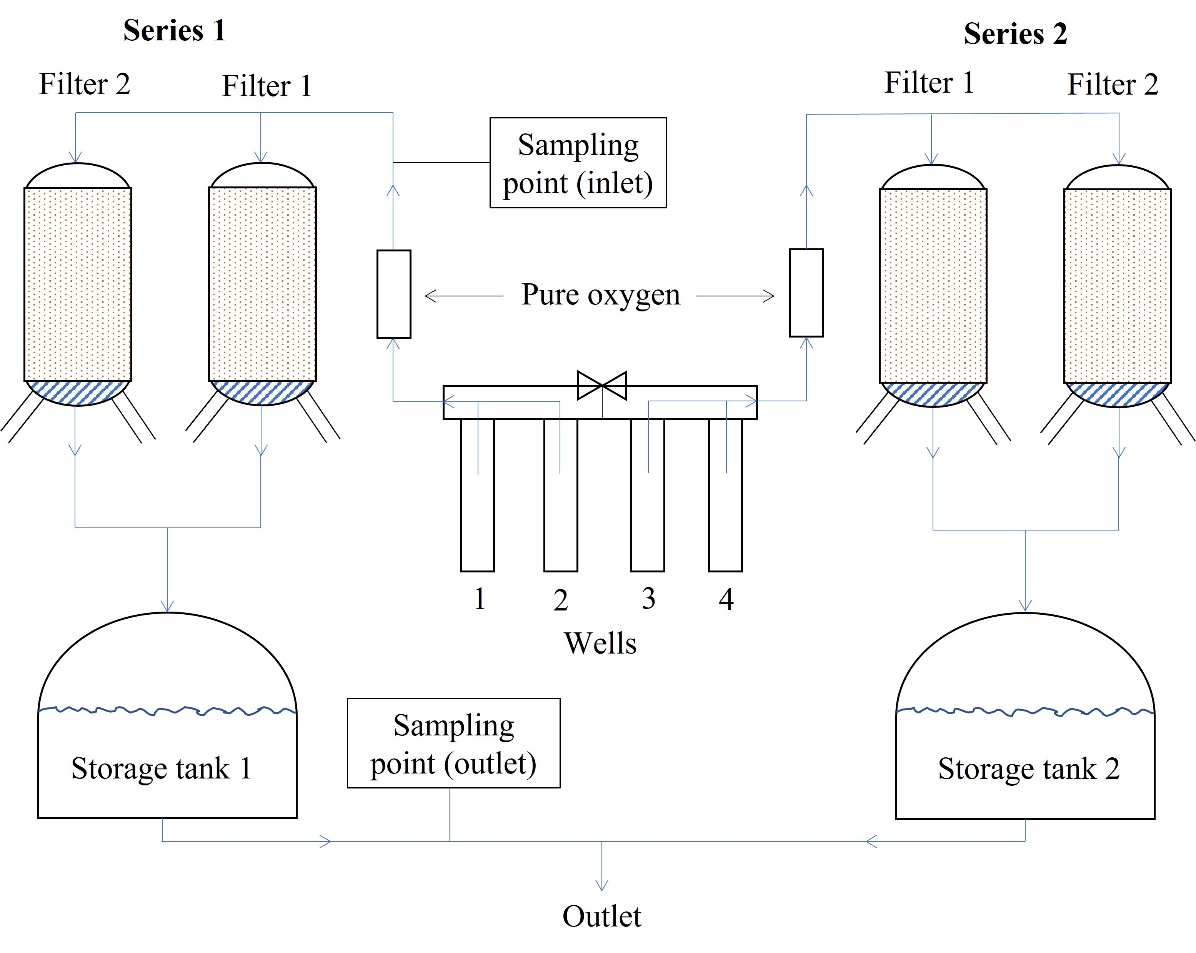


Figure S1. Scheme of the investigated drinking water facility (not at scale).


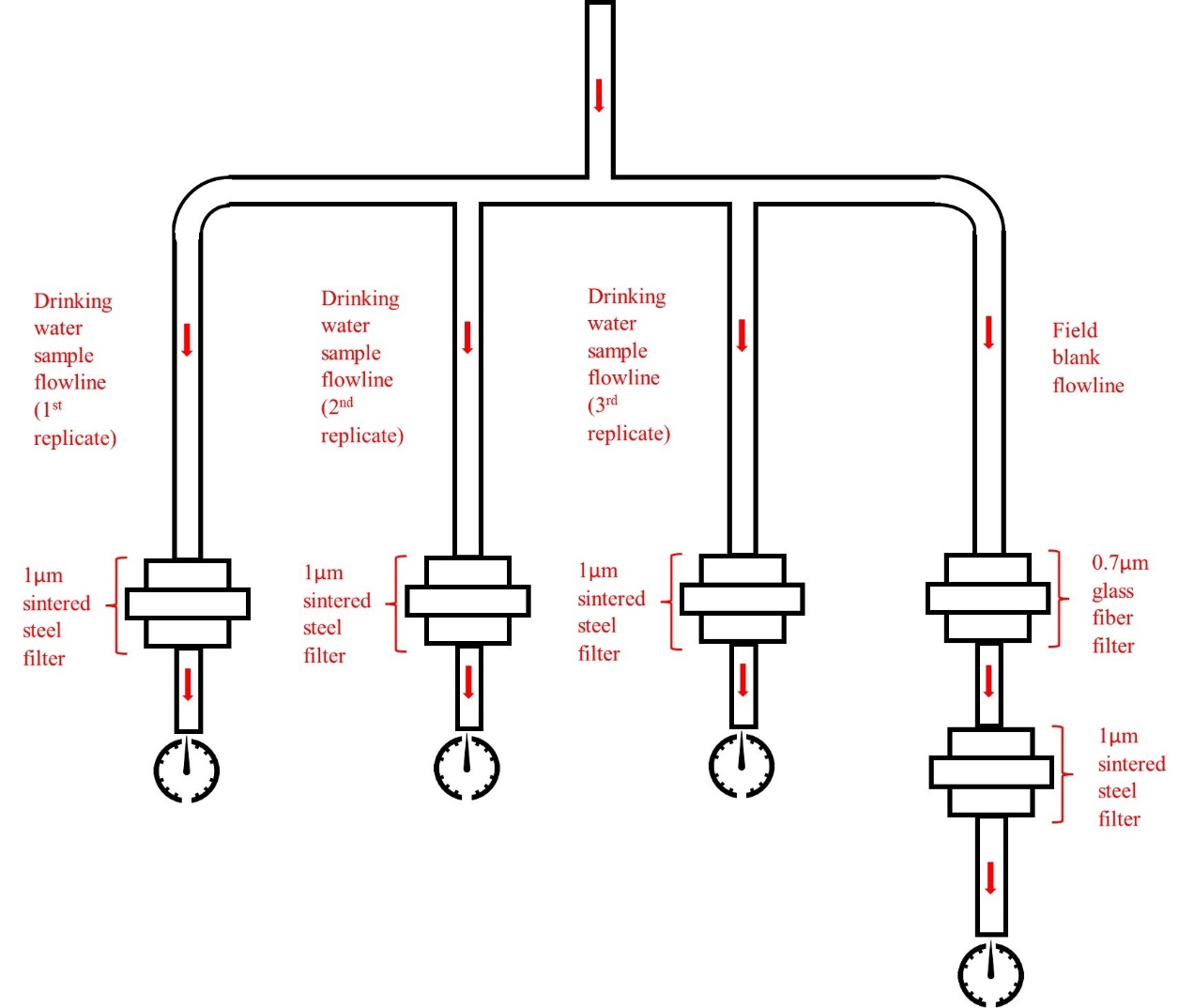

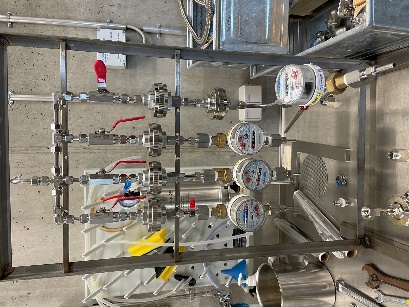


Figure S2. Scheme of the sampling device.

# Sample preparation and data post-processing with the siMPle software


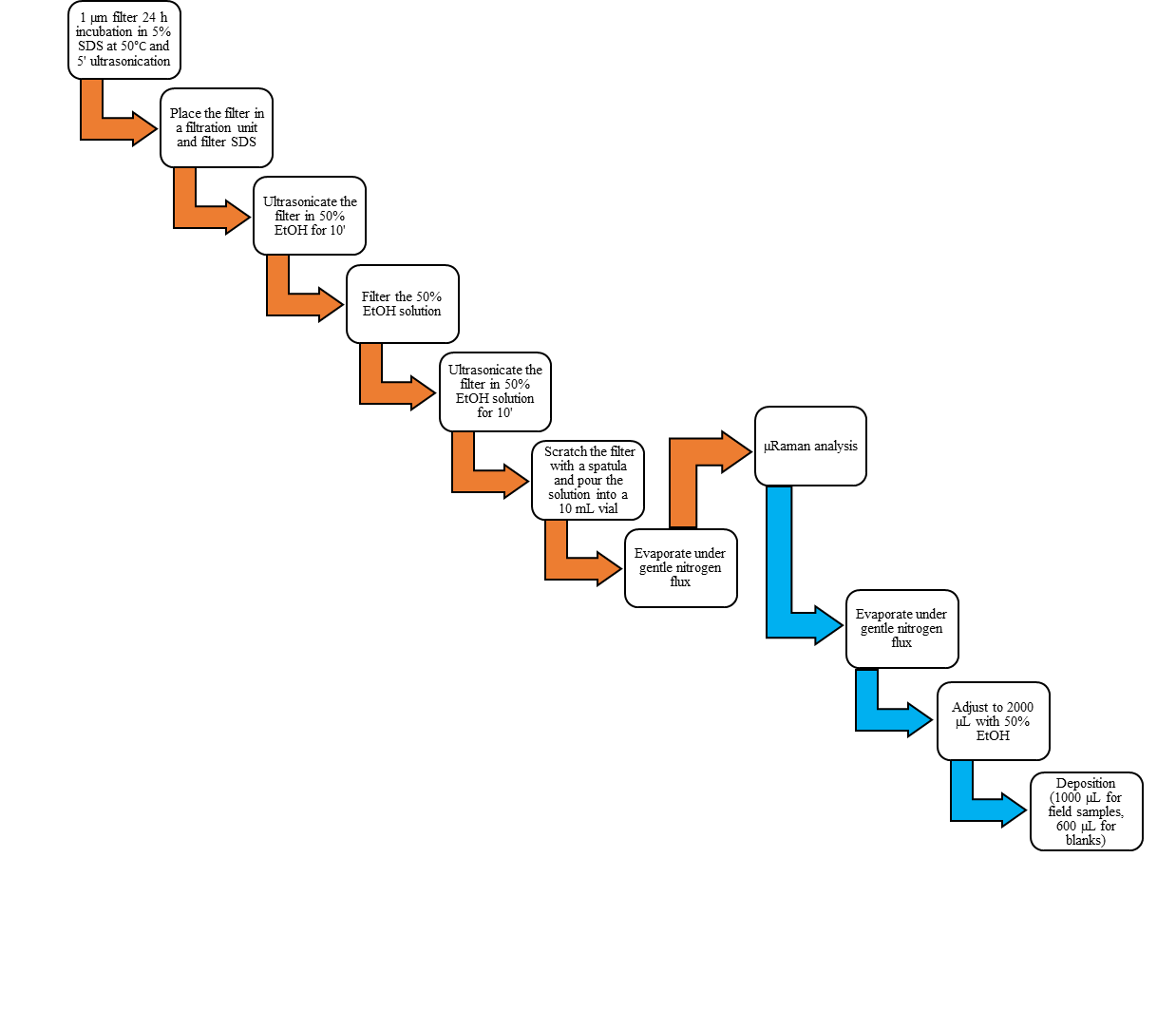
Figure S3 illustrates the overall sample preparation protocol performed before the µFTIR analysis.

Figure S3. Scheme of the cascade sample preparation protocol. The orange arrows indicate the steps performed before the µRaman analysis, while the blue ones are the steps prior to the µFTIR analysis.

Each filter was incubated in 5% Sodium Dodecylsulphate (SDS, VWR, Germany) at 50°C for 24 hours in a thermal bath (Thermo Fisher Scientific, Germany) inside a clean beaker, then the particle–enriched SDS solution was filtered through the same filter in a glass filtering system connected to a vacuum pump. The glass components in direct contact with the filter were thoroughly rinsed with 50% ethanol (EtOH for HPLC, Th. Geyer GmbH, Germany), letting the solution percolate in a clean beaker. Then, the filter was removed, put in the same beaker, and covered with a layer of 50% EtOH. The beaker was covered with aluminum foil to prevent external contamination and ultra-sonicated for 10 minutes. A new glass filtering unit was equipped with the same filter and the particle–enriched 50% EtOH solution was filtered through it.

The filter was removed and put in a clean beaker. The glass parts of the system were rinsed again with 50% EtOH, recovering the solution in the same beaker, until the filter was covered with a layer of 50% EtOH. Then, the beaker was covered with aluminum foil and ultra-sonicated for 10 minutes to detach the particles still attached to the filter's surfaces. Finally, the filter was scratched with a clean steel spatula and rinsed with a small amount of 50% ethanol to maximize the particle recovery in the ethanolic solution.

The particle-enriched EtOH solution was poured into a clean 10 mL glass vial. The vial was finally put in an evaporator and kept under gentle nitrogen flow in a water bath (TurboVap Biotage, Sweden) at 55°C until exhaustion of the entire volume. Each dried sample was kept in the fridge until the µRaman investigation was performed, then the µFTIR analysis followed.

Figure S4 shows a compression cell. The ZnSe window is placed in the window holder in the middle of the metallic main body, then the metallic funnel (Ø ~ 10 mm) is put onto the window. Finally, the system is sealed by manually screwing the metallic ring around the window holder, and the deposition can take place.


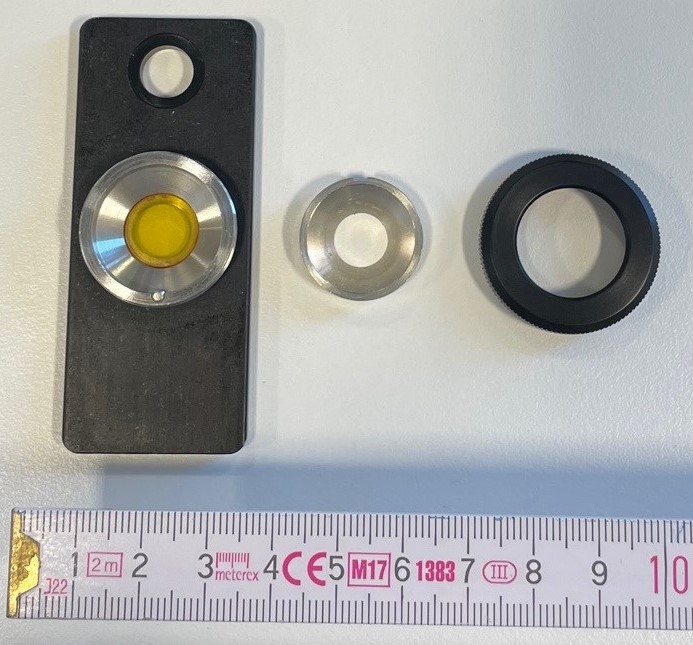

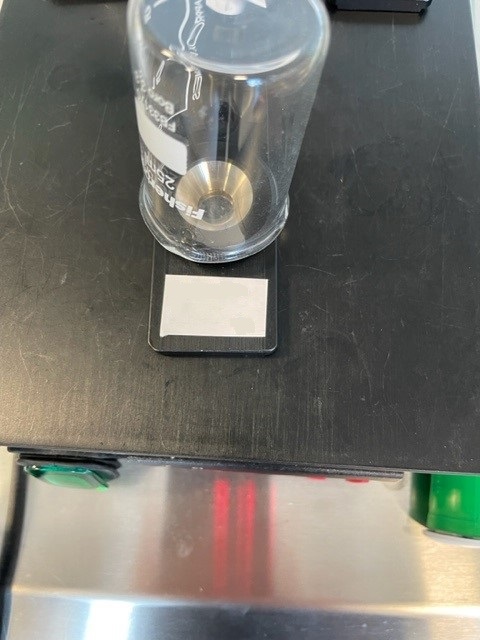


*a) b)*

Figure S4. a) A disassembled compression cell (notice the yellow ZnSe window in the center); b) A sample drying after the deposition.

Figure S5 shows an example of a dried active area before the µFTIR analysis (Ø ~ 10 mm).


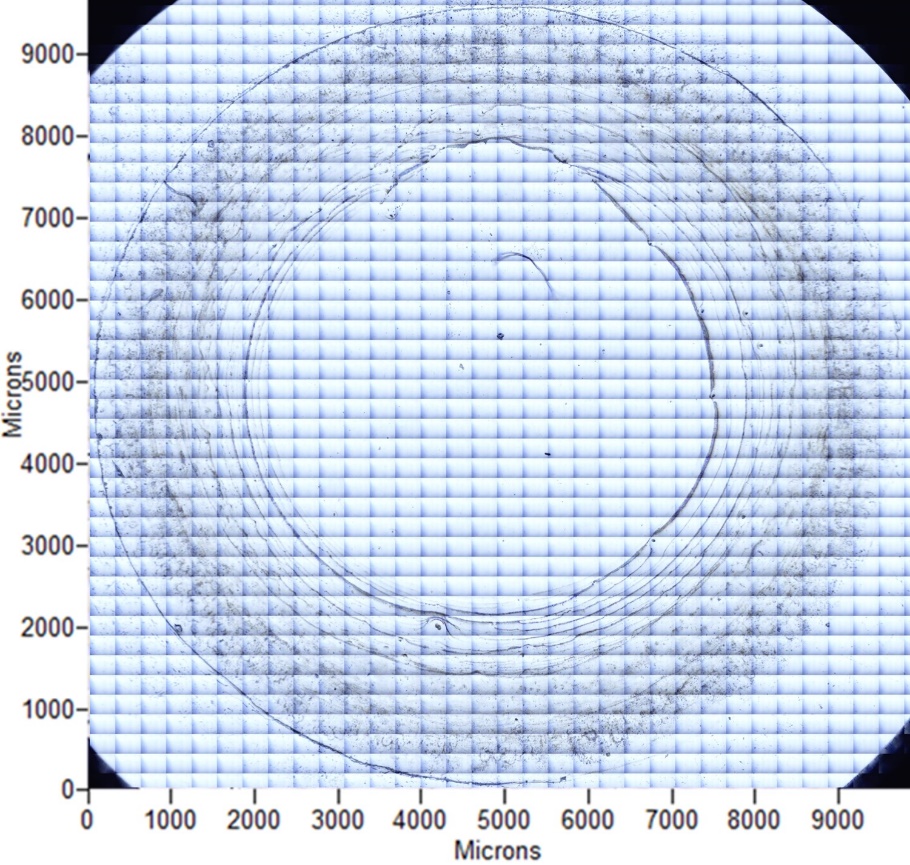


Figure S5. Example of visible montage of a dried active area before the µFTIR analysis.

Figures S6 – S8 are visible images of some of the MPs found in the samples.


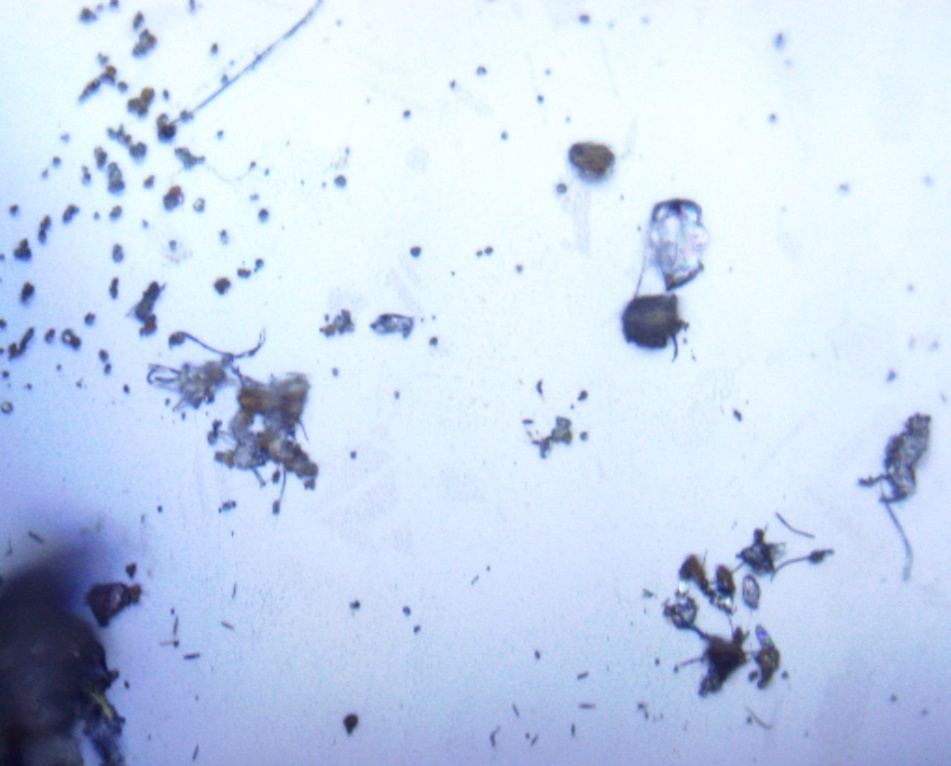


50 µm

Figure S6. Visible image of fragments and fibres taken prior to the analysis (25x).


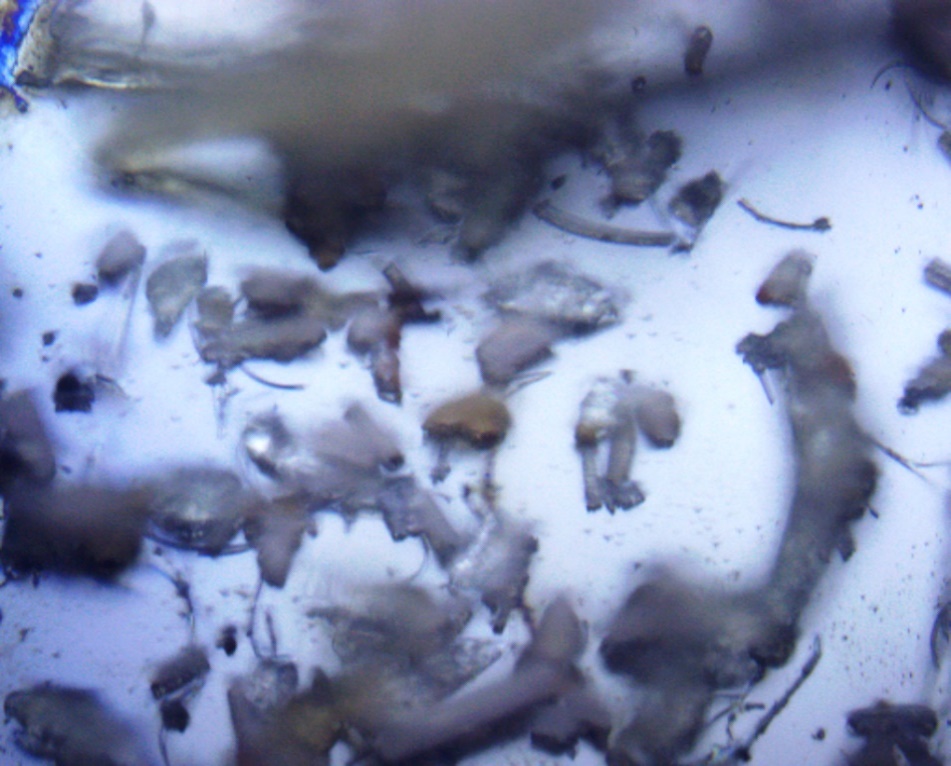


50 µm

Figure S7. Visible image of fragments and fibres taken prior to the analysis (25x).


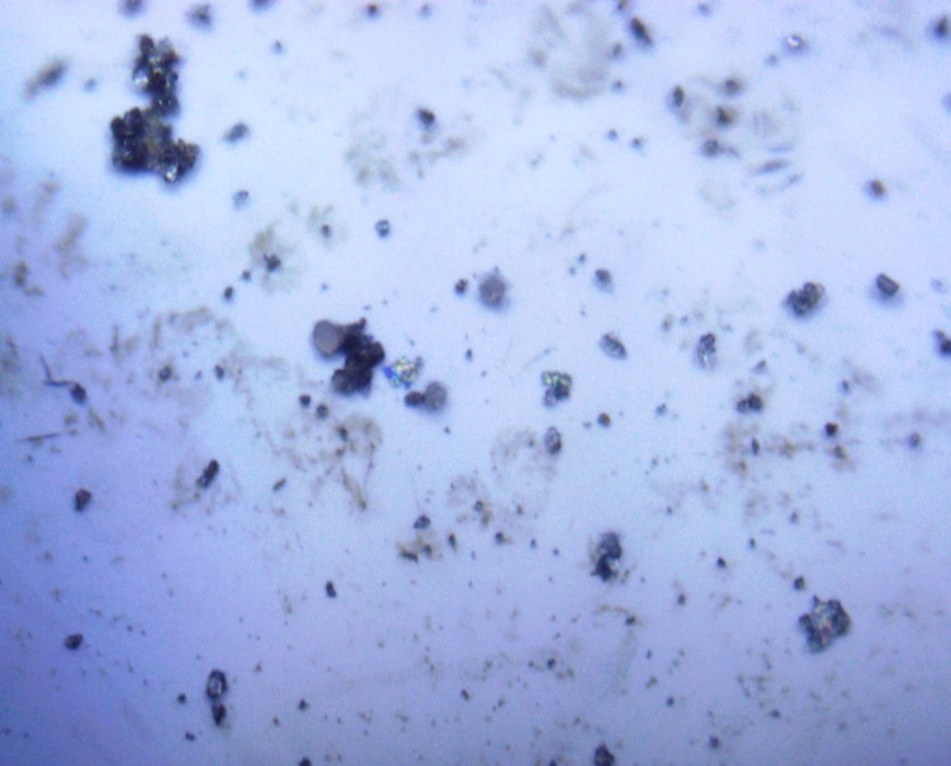


50 µm

Figure S8. Visible image of fragments taken prior to the analysis (25x).

siMPle v. 1.3.1β was employed for µFTIR data post-processing. Figure S9a) is an example of an experimental heatmap and Figure S9b) represents the chemical map obtained after the spectral matching. The spectral identification is performed for each pixel of the heatmap, by comparing the zero-order and the first-order derivative of the experimental spectrum and the reference from the library. Then, the software automatically assigns the experimental spectrum to one of the library’s polymeric groups according to the Pearson coefficient estimated from the matching.

*
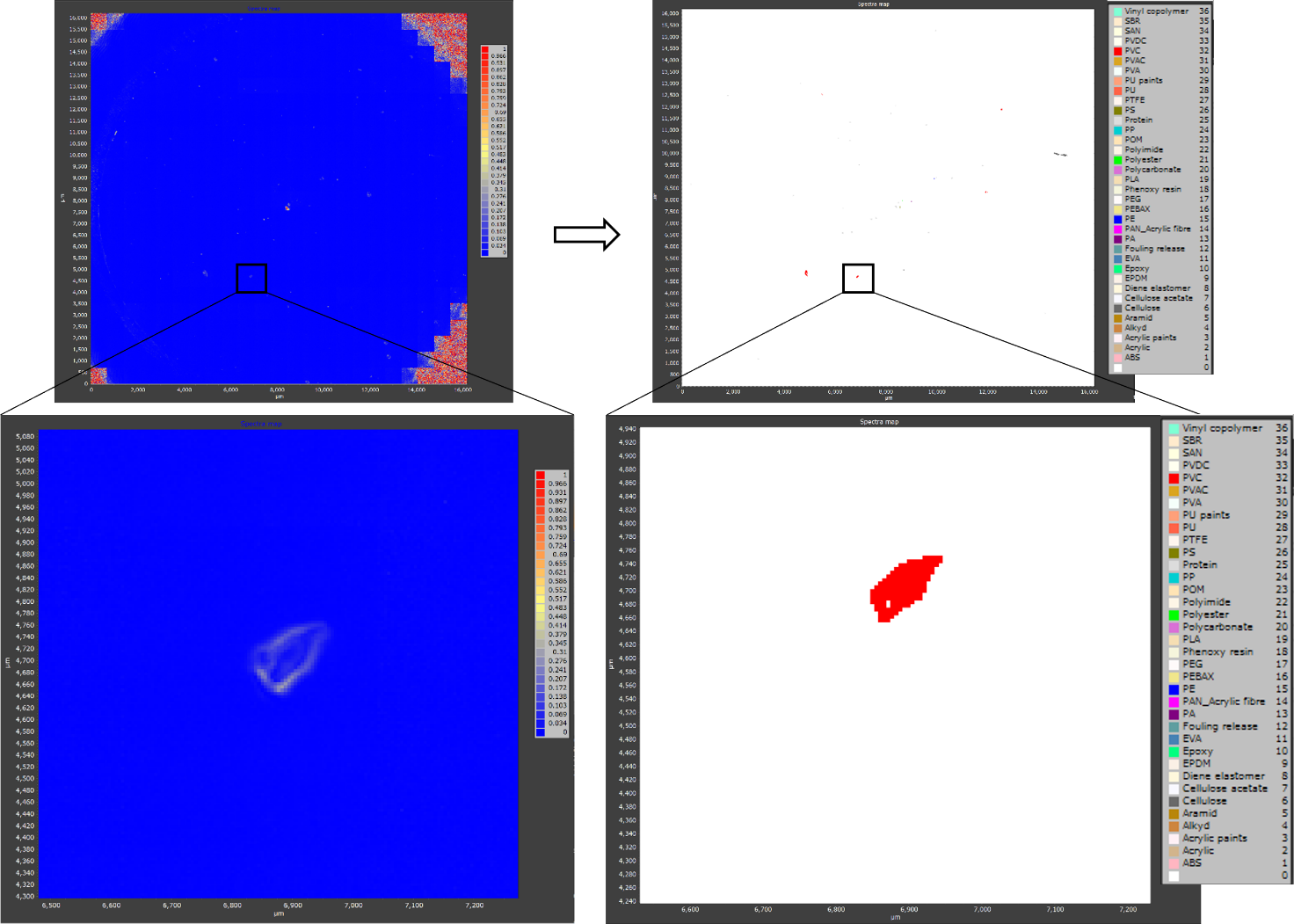
a) b)*

Figure S9. a) Example of an experimental heatmap. The color scale indicates the IR absorbance of the different regions in the sample; b) Experimental chemical map obtained after the spectral matching of each pixel with the reference spectra in the library. The matching quality is estimated through the Pearson coefficient and the polymeric group is assigned accordingly. The particle shown was recognized as PVC.

# Morphological analysis with µFTIR

Table S1 show the frequency of the MPs according to the five chosen length ranges.

| **Inlet frequency (%)** | | | | | |
| --- | --- | --- | --- | --- | --- |
| **Day** | **6.6 – 50 µm** | **50 – 100 µm** | **100 – 200 µm** | **200 – 500 µm** | **500+ µm** |
| 1 | 59.65 | 31.58 | 5.26 | 1.75 | 1.75 |
| 2 | 36.67 | 40.00 | 10.00 | 10.00 | 3.33 |
| 3 | 46.43 | 25.00 | 25.00 | 3.57 | 0.00 |
| 4 | 33.33 | 36.36 | 19.70 | 9.09 | 1.52 |
| 5 | 25.49 | 47.06 | 21.57 | 1.96 | 3.92 |
| **Mean** | 40.09 | 36.64 | 15.95 | 5.17 | 2.15 |
| **Outlet frequency (%)** | | | | | |
| **Day** | **6.6 – 50 µm** | **50 – 100 µm** | **100 – 200 µm** | **200 – 500 µm** | **500+ µm** |
| 1 | 34.48 | 31.03 | 24.14 | 6.90 | 3.45 |
| 2 | 25.00 | 45.00 | 20.00 | 10.00 | 0.00 |
| 3 | 13.33 | 40.00 | 26.67 | 13.33 | 6.67 |
| 4 | 31.25 | 37.50 | 31.25 | 0.00 | 0.00 |
| 5 | 23.81 | 33.33 | 33.33 | 9.52 | 0.00 |
| **Mean** | 26.73 | 36.63 | 26.73 | 7.92 | 1.98 |

Table S1. MP length ranges frequency over Days 1 – 5 at the waterworks' inlet and outlet with mean values. Values not corrected for blank contamination.

Figure S10 is a boxplot graphic of the MP length over Days 1 – 5 at the waterworks' inlet and outlet.


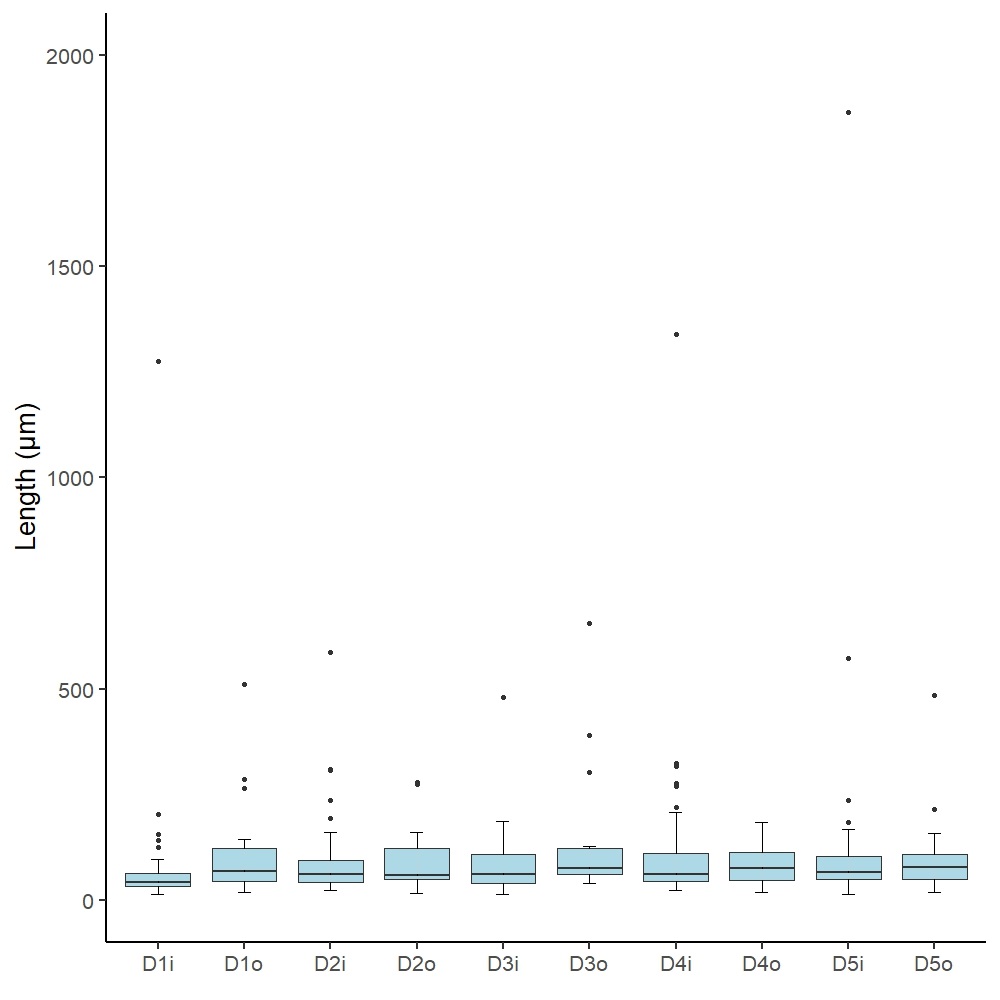
Figure S10. Boxplot of the MP length over Days 1 – 5 at the waterworks' inlet and outlet. The black line in the boxes represents the median and the points the outliers. Values not corrected for blank contamination.

Figure S11 reports the counts and overall frequency of fibres and fragments. MPs with a length/width ratio greater or equal to 3 were classified as fibres, otherwise fragments.


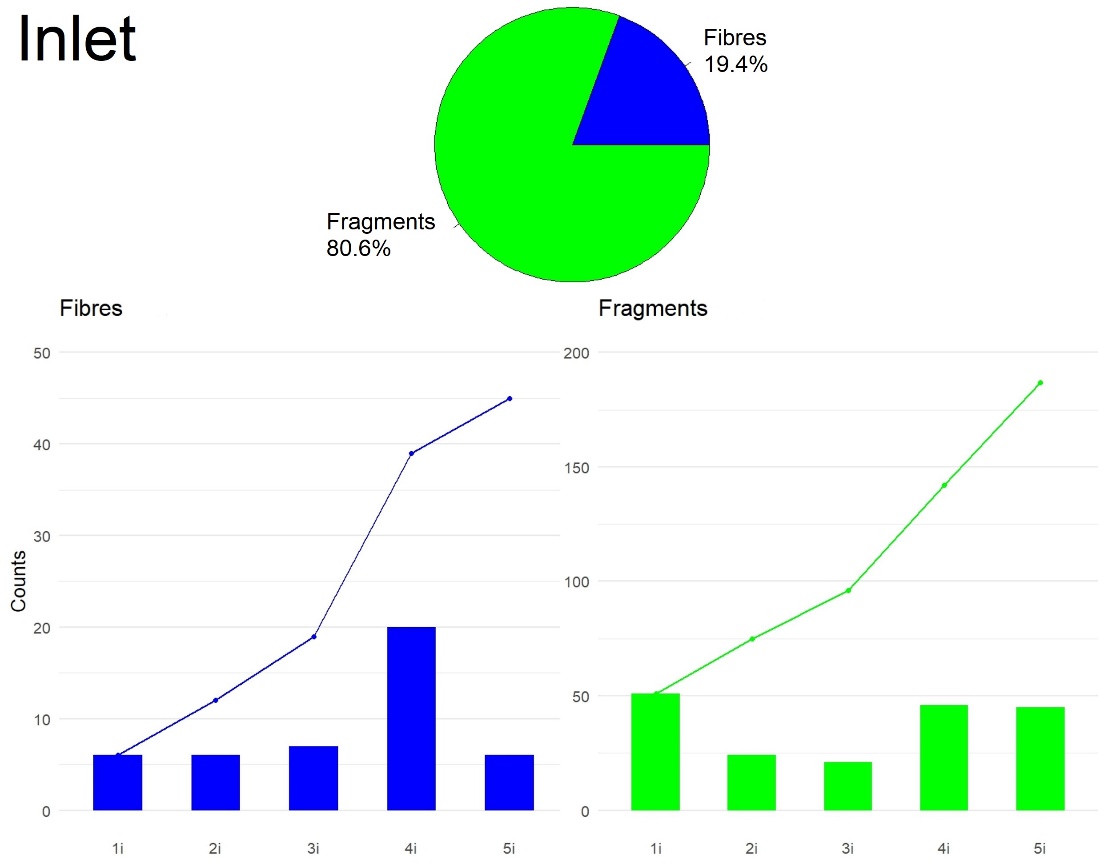


*
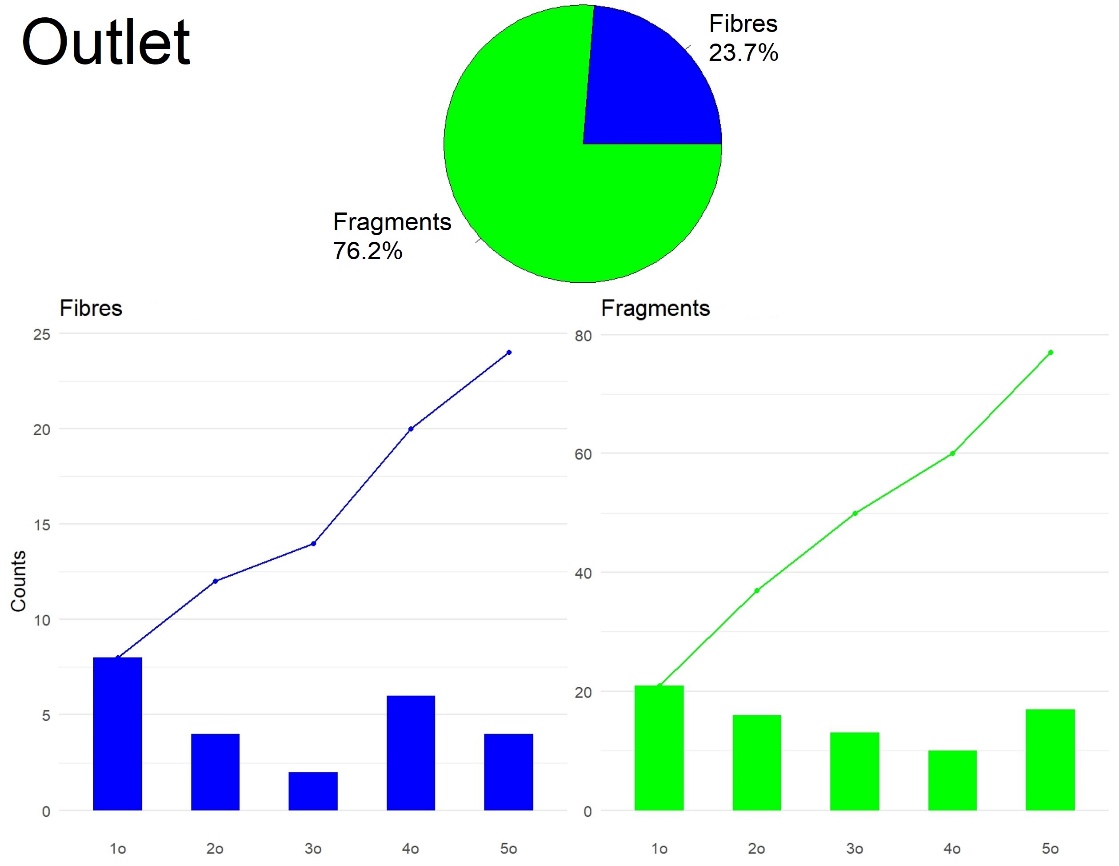
*

Figure S11. Counts of fibres and fragments at the inlet and outlet over the investigated period. The lines indicate the cumulated counts. Values not corrected for blank contamination.

# Microplastic quantification with µFTIR

The following plastic groups were identified with the µFTIR analysis: ABS (acrylonitrile-butadiene-styrene copolymer), acrylic (poly-acrylic), alkyd, epoxy (epoxy resins), Fouling release, PA (poly-amide), PE (poly-ethylene), PET (poly-ester), POM (poly-oxymethylene), PP (poly-propylene), PS (poly-styrene), PTFE (poly-tetrafluoroethylene), PVC (poly-vinylchloride), and PVDC (poly-vinylidenechloride). The "Other" category gathers the following polymers: ABS, alkyd, epoxy, fouling release, PA, POM, PP, and PTFE, whilst PVC and PVDC were unified under the category PV(D)C. Table S2 summarizes the MP counts and mass abundance over the investigated period at the waterworks' inlet and outlet.

| **Sample** | **MP abundance (N/m^3^)** | **MP abundance**  **(µg/m^3^)** |
| --- | --- | --- |
| D1i | 33.54 ± 6.07 | 7.04 ± 13.09 |
| D1o | 8.44 ± 5.81 | 3.25 ± 3.98 |
| D2i | 16.75 ± 27.26 | 6.23 ± 4.92 |
| D2o | 7.20 ± 4.25 | 0.79 ± 0.94 |
| D3i | 14.61 ± 14.90 | 0.34 ± 0.49 |
| D3o | 6.27 ± 3.64 | 8.07 ± 16.05 |
| D4i | 62.84 ± 104.37 | 258.88 ± 171.59 |
| D4o | 3.07 ± 0.92 | 0.53 ± 0.73 |
| D5i | 31.57 ± 16.97 | 833.55 ± 556.29 |
| D5o | 9.84 ± 5.37 | 1.40 ± 2.54 |
| **Mean inlet** | 31.86 ± 17.17 | 221.21 ± 320.04 |
| **Mean outlet** | 6.96 ± 2.27 | 2.81 ± 2.78 |

Table S2. MP abundance according to the counts and mass estimates at the waterworks' inlet and outlet over the five investigated days (µFTIR dataset).

Table S3 summarizes the removal efficiency data cumulated over the five investigated days.

| **Day** | **Counts removal efficiency (%)** | **Mass removal efficiency (%)** |
| --- | --- | --- |
| 1 | 74.83 ± 17.32 | 53.83 ± 56.54 |
| 2+1 | 68.90 ± 25.39 | 69.55 ± 15.18 |
| 3+2+1 | 66.24 ± 24.90 | 11.02 ± 15.18 |
| 4+3+2+1 | 80.44 ± 1.47 | 95.36 ± 0.83 |
| 5+4+3+2+1 | 78.14 ± 49.70 | 98.73 ± 11.10 |

Table S3. MPs removal efficiency over the five investigated days with mean values (µFTIR dataset).


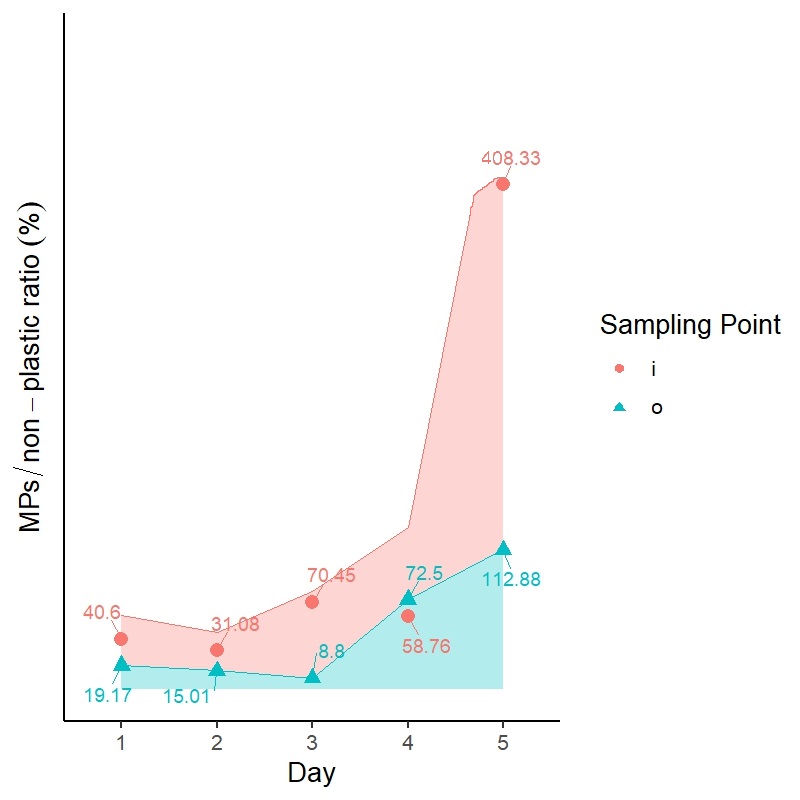
Figure S12 shows the MPs/non-plastic ratio at the waterworks' inlet and outlet in the investigated period as per the µFTIR analysis.

Figure S12. MPs/non-plastic ratio over Days 1 – 5 at the waterworks' inlet and outlet (µFTIR).

Tables S4 and S5 report on the MP frequency at the inlet and outlet according to the MP counts and MP estimated mass, respectively.

| **Inlet frequency (%)** | | | | | | |
| --- | --- | --- | --- | --- | --- | --- |
| **Day** | **Acrylic** | **PE** | **PET** | **PS** | **PV(D)C** | **Other** |
| 1 | 19.15 | 24.34 | 5.35 | 2.03 | 41.10 | 8.02 |
| 2 | 60.43 | 13.97 | 13.94 | 4.67 | 6.99 | 0.00 |
| 3 | 0.00 | 13.64 | 0.00 | 0.00 | 31.16 | 55.19 |
| 4 | 24.15 | 42.48 | 2.77 | 0.00 | 28.75 | 1.85 |
| 5 | 32.15 | 0.00 | 8.61 | 5.19 | 54.04 | 0.00 |
| **Mean** | 26.20 | 21.78 | 5.69 | 2.19 | 34.82 | 9.31 |
| **Outlet frequency (%)** | | | | | | |
| **Day** | **Acrylic** | **PE** | **PET** | **PS** | **PV(D)C** | **Other** |
| 1 | 0.00 | 12.38 | 39.30 | 9.23 | 26.70 | 12.38 |
| 2 | 0.00 | 0.00 | 46.46 | 0.00 | 25.36 | 28.18 |
| 3 | 0.00 | 20.48 | 70.84 | 0.00 | 0.00 | 8.68 |
| 4 | 0.00 | 0.00 | 0.00 | 0.00 | 71.42 | 28.58 |
| 5 | 5.56 | 0.00 | 0.00 | 0.00 | 84.73 | 9.71 |
| **Mean** | 1.20 | 6.89 | 33.58 | 2.94 | 39.00 | 16.37 |

Table S4. MP polymer frequency over the five investigated days at the waterworks' inlet and outlet with mean values (counts).

| **Inlet frequency (%)** | | | | | | |
| --- | --- | --- | --- | --- | --- | --- |
| **Day** | **Acrylic** | **PE** | **PET** | **PS** | **PV(D)C** | **Other** |
| 1 | 39.47 | 2.26 | 0.00 | 0.04 | 58.22 | 0.00 |
| 2 | 4.83 | 2.49 | 57.06 | 0.02 | 35.59 | 0.00 |
| 3 | 5.36 | 15.21 | 0.00 | 0.00 | 59.19 | 20.24 |
| 4 | 0.09 | 0.36 | 98.79 | 0.00 | 0.74 | 0.01 |
| 5 | 0.02 | 0.00 | 99.61 | < 0.01 | 0.36 | 0.00 |
| **Mean** | 0.50 | 0.14 | 97.90 | < 0.01 | 1.45 | < 0.01 |
| **Outlet frequency (%)** | | | | | | |
| **Day** | **Acrylic** | **PE** | **PET** | **PS** | **PV(D)C** | **Other** |
| 1 | 0.00 | 0.26 | 79.76 | 0.06 | 8.93 | 10.99 |
| 2 | 0.00 | 0.00 | 83.68 | 0.00 | 10.66 | 5.65 |
| 3 | 0.00 | 0.40 | 99.19 | 0.00 | 0.38 | 0.03 |
| 4 | 0.00 | 0.00 | 0.00 | 0.00 | 100.00 | 0.00 |
| 5 | 0.19 | 0.00 | 57.09 | 0.00 | 26.62 | 16.10 |
| **Mean** | 0.01 | 0.31 | 88.72 | 0.01 | 6.88 | 4.05 |

Table S5. MP polymer frequency over the five investigated days at the waterworks' inlet and outlet with mean values (mass estimate).

Table S6 and Table S7 summarize the mean MP amount in the drinking water samples (not blank corrected) and field blanks, respectively, as counts of particles (N) and mass of particles (µg), for each identified plastic polymer. The values in Tables S6 and S7 were obtained by normalizing the MP counts or estimated mass in the active area (deposition of 1000 µL for drinking water samples and 600 µL for field blanks) by the total volume after reconstitution (2000 µL). The MP counts were also rounded to give an integer.

| **Polymer** | **Raw MP counts per polymer (N/m^3^)** | | | | | | | | | | |
| --- | --- | --- | --- | --- | --- | --- | --- | --- | --- | --- | --- |
|  | **D1i** | **D1o** | **D2i** | **D2o** | **D3i** | **D3o** | **D4i** | **D4o** | **D5i** | **D5o** | **Blank mean** |
| ABS | 0 | 0 | 0 | 0 | 0 | 0 | 1 | 0 | 0 | 0 | 0 |
| Acrylic | 5 | 0 | 9 | 0 | 5 | 0 | 9 | 0 | 8 | 1 | 0 |
| Alkyd | 0 | 0 | 0 | 0 | 0 | 0 | 15 | 0 | 0 | 0 | 1 |
| Epoxy | 0 | 0 | 0 | 0 | 0 | 0 | 0 | 0 | 0 | 1 | 0 |
| Fouling release | 0 | 1 | 0 | 0 | 0 | 0 | 0 | 0 | 0 | 0 | 0 |
| PA | 0 | 1 | 0 | 0 | 0 | 0 | 0 | 0 | 0 | 0 | 0 |
| PE | 8 | 2 | 2 | 0 | 0 | 0 | 15 | 0 | 0 | 0 | 0 |
| PET | 5 | 5 | 2 | 5 | 1 | 5 | 3 | 0 | 3 | 2 | 4 |
| POM | 0 | 0 | 0 | 0 | 0 | 1 | 0 | 0 | 0 | 0 | 0 |
| PP | 4 | 1 | 1 | 3 | 5 | 0 | 0 | 0 | 1 | 0 | 4 |
| PS | 1 | 1 | 1 | 0 | 0 | 0 | 0 | 0 | 1 | 0 | 0 |
| PTFE | 0 | 0 | 0 | 1 | 0 | 0 | 0 | 1 | 0 | 0 | 0 |
| PVC | 11 | 6 | 5 | 3 | 5 | 1 | 13 | 5 | 16 | 7 | 5 |
| PVDC | 4 | 2 | 1 | 1 | 1 | 1 | 3 | 4 | 5 | 4 | 2 |

Table S6. MP counts per m^3^ (N/m^3^) for each identified plastic polymer (µFTIR dataset). Values for the drinking water samples were not blank corrected.

| **Polymer** | **Raw MP mass per polymer (µg/m^3^)** | | | | | | | | | | |
| --- | --- | --- | --- | --- | --- | --- | --- | --- | --- | --- | --- |
|  | **D1i** | **D1o** | **D2i** | **D2o** | **D3i** | **D3o** | **D4i** | **D4o** | **D5i** | **D5o** | **Blank mean** |
| ABS | 0 | 0 | 0 | 0 | 0 | 0 | 0.02 | 0 | 0 | 0 | 0 |
| Acrylic | 2.95 | 0 | 0.45 | 0 | 0.21 | 0 | 0.19 | 0 | 0.14 | 0.01 | 0 |
| Alkyd | 0 | 0 | 0 | 0 | 0 | 0 | 0 | 0 | 0 | 0 | 0.07 |
| Epoxy | 0 | 0 | 0 | 0 | 0 | 0 | 0 | 0 | 0 | 0.02 | 0 |
| Fouling release | 0 | 0.02 | 0 | 0 | 0 | 0 | 0 | 0 | 0 | 0 | 0 |
| PA | 0 | 0.02 | 0 | 0 | 0 | 0 | 0 | 0 | 0 | 0 | 0 |
| PE | 0.23 | 0.03 | 0.23 | 0 | 0.02 | 0.17 | 0.73 | 0 | 0 | 0 | 0 |
| PET | 0.14 | 4.20 | 2.22 | 1.47 | 0.10 | 15.62 | 68.05 | 0 | 192.23 | 1.15 | 1.29 |
| POM | 0 | 0 | 0 | 0 | 0 | 0.01 | 0 | 0 | 0 | 0.59 | 0 |
| PP | 0.04 | 0.46 | < 0.01 | 0.13 | 0.10 | 0 | 0 | 0 | < 0.1 | 0 | 0.29 |
| PS | < 0.01 | 0.01 | < 0.01 | 0 | 0 | 0 | 0 | 0 | 0.02 | 0 | 0 |
| PTFE | 0 | 0 | 0 | 0.06 | 0 | 0 | 0 | 0.01 | 0 | 0 | 0 |
| PVC | 1.66 | 0.99 | 2.64 | 0.49 | 0.25 | 0.19 | 2.03 | 1.00 | 2.43 | 0.76 | 0.45 |
| PVDC | 0.02 | 0.02 | 0.02 | < 0.01 | < 0.01 | 0.04 | 0.02 | 0.03 | 0.17 | 0.15 | 0.02 |

Table S7. MP mass per m^3^ (µg/m^3^) for each identified plastic polymer (µFTIR dataset). Values for the drinking water samples were not blank corrected.

# Microplastic µFTIR spectra


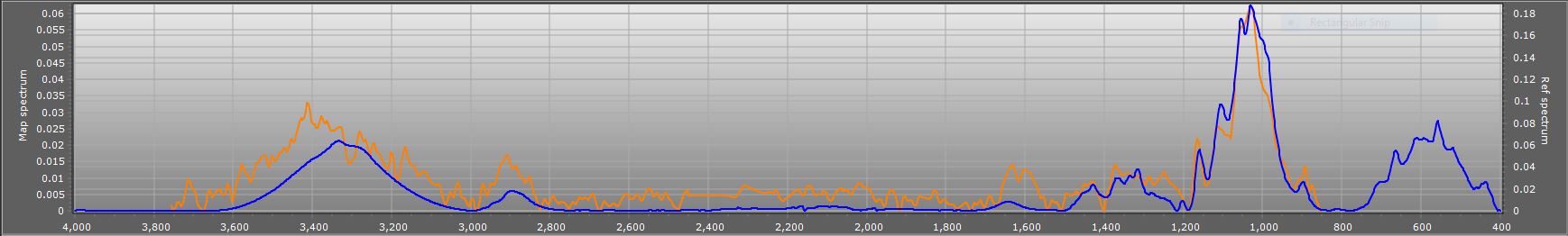
Figures S13 – S24 are examples of experimental spectra of the identified polymer types (siMPle dashboard).

Figure S13. µFTIR spectrum of Cellulose (experimental spectrum is in orange, reference in blue).

*
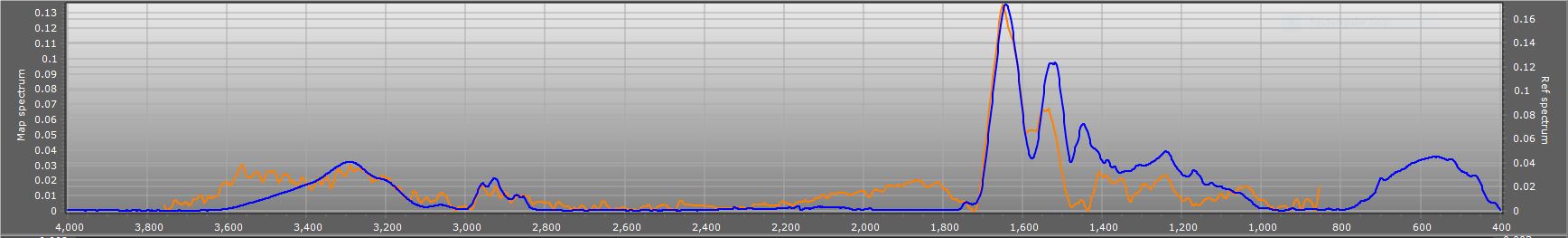
Figure S14. µFTIR spectrum of Zein, a vegetal protein (experimental spectrum is in orange, reference in blue).*

*
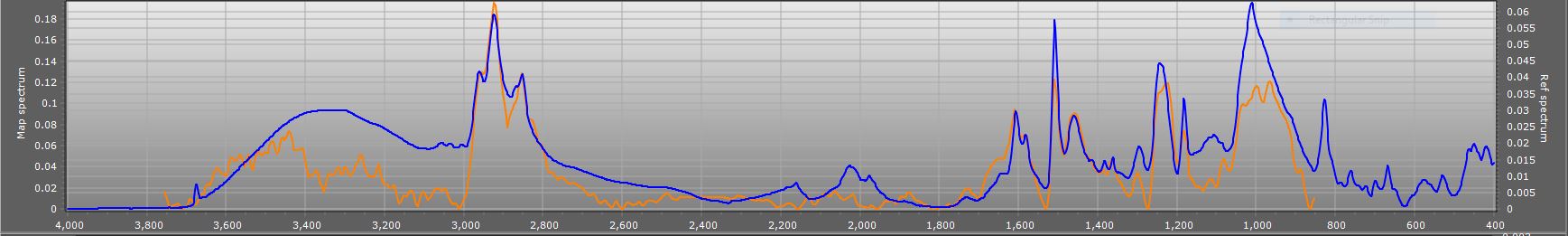

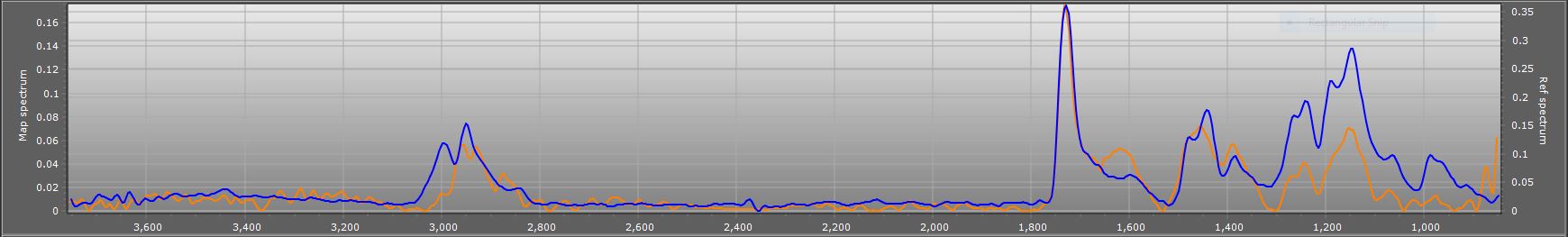
Figure S15. µFTIR spectrum of PMMA, named Acrylic in the discussion (experimental spectrum is in orange, reference in blue).*

*
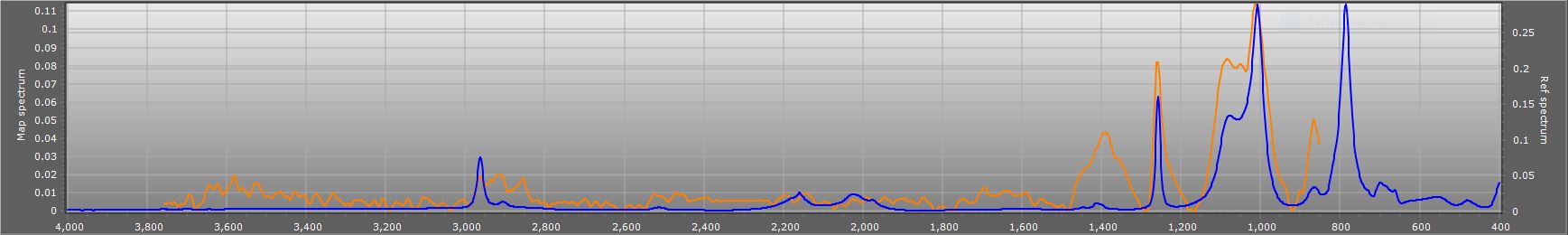
Figure S16.* *µFTIR spectrum of Epoxy resin, named Epoxy in the discussion (experimental spectrum is in orange, reference in blue).*

Figure S17. µFTIR spectrum of Fouling release (experimental spectrum is in orange, reference in blue).


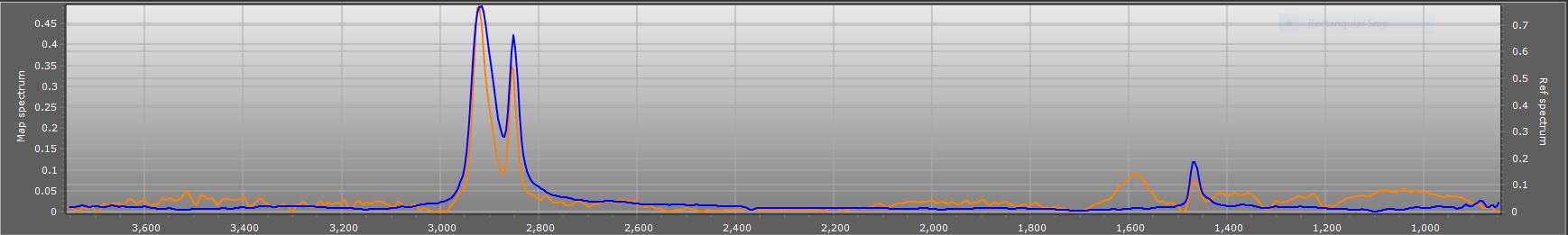
 Figure S18. µFTIR spectrum of PE (experimental spectrum is in orange, reference in blue).

*
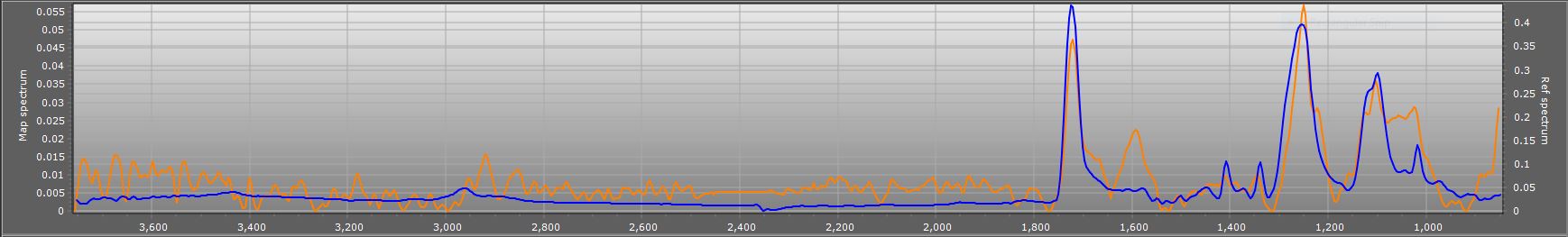
*


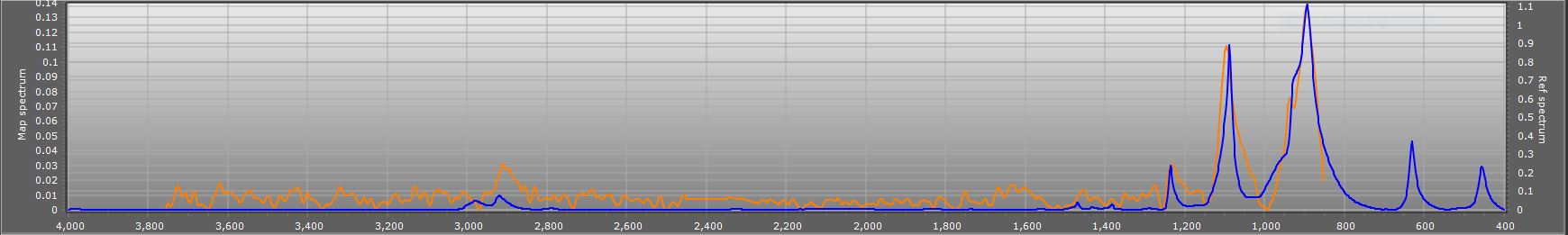
Figure S19. µFTIR spectrum of PET (experimental spectrum is in orange, reference in blue).

Figure S20. µFTIR spectrum of POM (experimental spectrum is in orange, reference in blue).


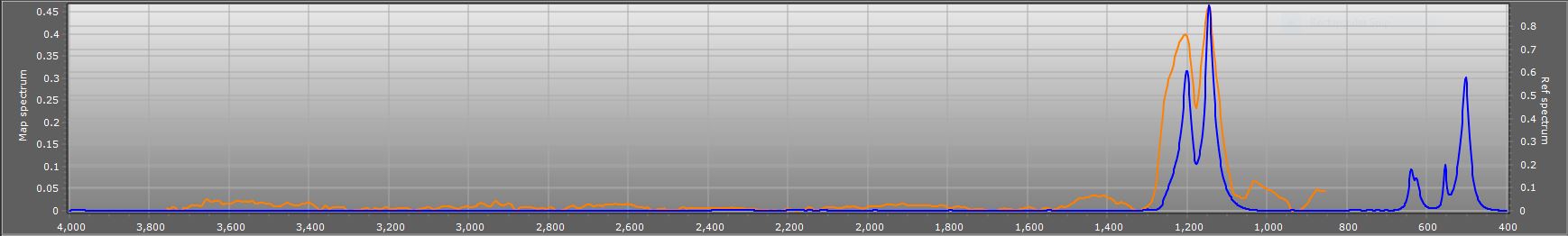
Figure S21. µFTIR spectrum of PP (experimental spectrum is in orange, reference in blue).


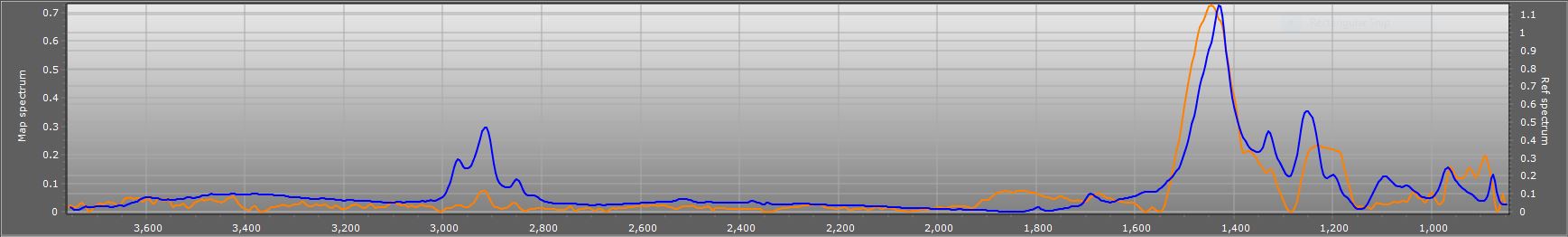
Figure S22. µFTIR spectrum of PTFE (experimental spectrum is in orange, reference in blue).

Figure S23. µFTIR spectrum of PVC (experimental spectrum is in orange, reference in blue).


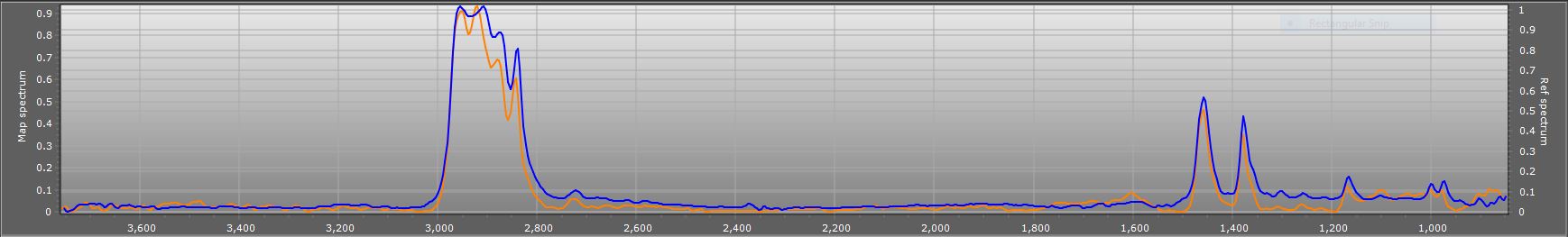


Figure S24. µFTIR spectrum of PVDC (experimental spectrum is in orange, reference in blue).


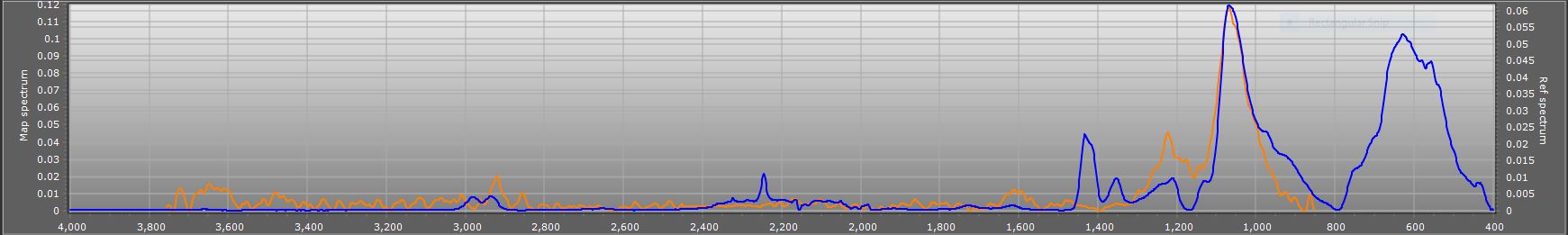


# References

Maurizi L., Iordachescu L., Kirstein I.V., Nielsen A.H., Vollertsen J., Do drinking water plants retain microplastics? An exploratory study using Raman micro-spectroscopy, Heliyon, 9, 6, e17113 (2023), ISSN 2405-8440, <https://doi.org/10.1016/j.heliyon.2023.e17113>
